# Genome-Wide Essentiality Analysis of *Mycobacterium abscessus* by Saturated Transposon Mutagenesis and Deep Sequencing

**DOI:** 10.1128/mBio.01049-21

**Published:** 2021-06-15

**Authors:** Dalin Rifat, Liang Chen, Barry N. Kreiswirth, Eric L. Nuermberger

**Affiliations:** a The Center for Tuberculosis Research, Department of Medicine, Johns Hopkins University, Baltimore, Maryland, USA; b Center for Discovery and Innovation, Hackensack Meridian Health, Nutley, New Jersey, USA; c Department of Medical Sciences, Hackensack Meridian School of Medicine, Nutley, New Jersey, USA; University of Massachusetts—Amherst

**Keywords:** *Mycobacterium abscessus*, Himar1 mutagenesis, transposon sequencing (Tn-Seq), essential gene, essentiality, deep sequencing, mycobacterium, essential gene, gene disruption, genetics, genomics, transposon

## Abstract

Mycobacterium abscessus is an emerging opportunistic human pathogen that naturally resists most major classes of antibiotics, making infections difficult to treat. Thus far, little is known about *M. abscessus* physiology, pathogenesis, and drug resistance. Genome-wide analyses have comprehensively catalogued genes with essential functions in Mycobacterium tuberculosis and Mycobacterium avium subsp. *hominissuis* (here, *M. avium*) but not in *M. abscessus*. By optimizing transduction conditions, we achieved full saturation of TA insertion sites with Himar1 transposon mutagenesis in the *M. abscessus* ATCC 19977^T^ genome, as confirmed by deep sequencing prior to essentiality analyses of annotated genes and other genomic features. The overall densities of inserted TA sites (85.7%), unoccupied TA sites (14.3%), and nonpermissive TA sites (8.1%) were similar to results in *M. tuberculosis* and *M. avium*. Of the 4,920 annotated genes, 326 were identified as essential, 269 (83%) of which have mutual homology with essential *M. tuberculosis* genes, while 39 (12%) are homologous to genes that are not essential in *M. tuberculosis* and *M. avium*, and 11 (3.4%) only have homologs in *M. avium*. Interestingly, 7 (2.1%) essential *M. abscessus* genes have no homologs in either *M. tuberculosis* or *M. avium*, two of which were found in phage-like elements. Most essential genes are involved in DNA replication, RNA transcription and translation, and posttranslational events to synthesize important macromolecules. Some essential genes may be involved in *M. abscessus* pathogenesis and antibiotics response, including certain essential tRNAs and new short open reading frames. Our findings will help to pave the way for better understanding of *M. abscessus* and benefit development of novel bactericidal drugs against *M. abscessus*.

## INTRODUCTION

Mycobacterium abscessus complex comprises the largest group of rapidly growing nontuberculosis mycobacteria (1). It causes chronic lung infection in individuals with cystic fibrosis (CF) and other structural lung diseases, as well as skin and skin structure infections (1–3). Treatment of *M. abscessus* lung infection is difficult. Cure rates are approximately 30% despite long courses of treatment with poorly tolerated regimens (4). The poor efficacy of existing treatments is attributable in part to the high degree of intrinsic resistance to most major classes of antibiotics, including most antituberculosis drugs (2, 5, 6). Safer, more effective drugs are urgently needed. Genome-wide essentiality analyses could help to identify new drug targets for development of novel drugs targeting *M. abscessus*.

*M. abscessus* is ubiquitous in the environment, including soil and water (4). Little knowledge exists regarding genome-wide elements that are essential for *M. abscessus* viability in the inanimate environment or its ability to cause disease. These knowledge gaps result in part from the limited availability and/or use of genetic tools (7–9). Comprehensive predictions of essential genes and other genetic elements, such as short open reading frames (ORFs), noncoding RNA, and tRNA in Mycobacterium tuberculosis and Mycobacterium avium subsp. *hominissuis* (here, *M. avium*), made using saturated transposon (Tn) mutant pools and deep sequencing were recently reported (10, 11). Transposon sequencing (Tn-Seq) is a powerful tool to determine the essentiality of genes or other genomic features for growth and survival under experimental conditions (10–13). Essential and conditionally essential genes may represent ideal targets for novel drugs or important virulence factors to target with interventions in order to better treat or prevent *M. abscessus* infections.

Here, we optimized conditions for Himar1 Tn mutagenesis of M. abscessus subsp. *abscessus* strain ATCC 19977^T^ to generate saturated Tn mutant pools and then prepared fully saturated DNA libraries for deep sequencing. Comprehensive genomic analysis was performed using a Hidden Markov Model (HMM) to predict essentiality of annotated genes and other genomic features for *in vitro* growth. We also compared essential genes of *M. abscessus* to those of *M. tuberculosis* and *M. avium* to characterize commonalities and differences in essentiality between those pathogens. Our findings provide insights for understanding *M. abscessus* pathogenesis and pave the way for developing safer, more effective drugs to treat *M. abscessus* infections.

## RESULTS

### Optimization of Tn mutagenesis.

**FIG 1:**
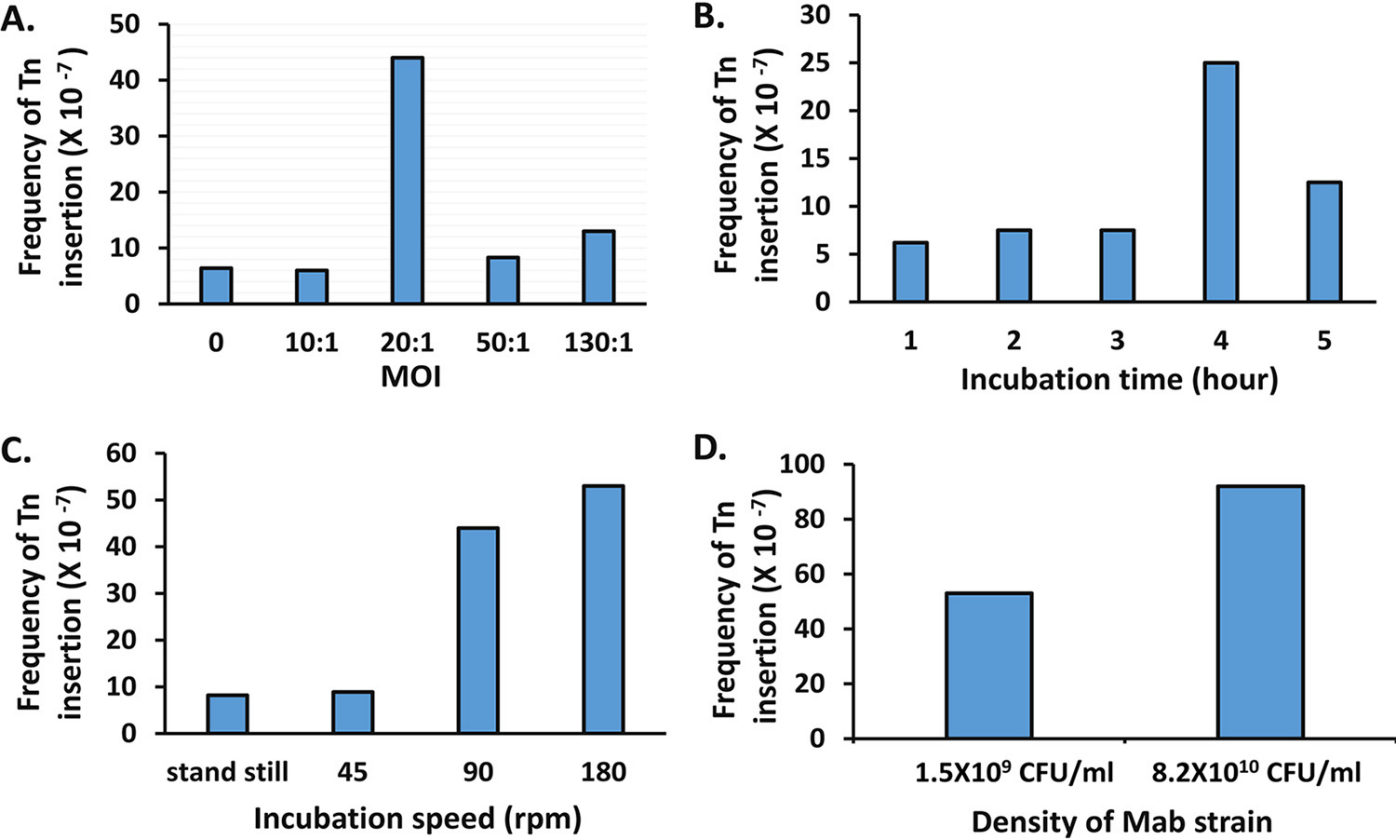
Optimization of experimental conditions for generation of a saturated Tn mutant pool using the Himar1 Tn. (A) A culture containing 1.5 × 10^9^ CFU/ml of *M. abscessus* ATCC 19977^T^ was infected at different MOIs (ratio of ΦmycomarT7 phage PFU to *M. abscessus* CFU counts) and incubated at 37°C in a shaker at 90 rpm for 3 h. (B) A culture containing 1.5 × 10^9^ CFU/ml of *M. abscessus* was infected with the same phage at an MOI of 10:1 and incubated at 37°C in a shaker at 90 rpm for different periods of time. (C) A culture containing 1.5 × 10^9^ CFU/ml of *M. abscessus* was infected with the same phage at an MOI of 20:1 and incubated at 37°C in a shaker at different speeds for 4 h. (D) Cultures containing different concentrations of *M. abscessus* were infected with the same phage at an MOI of 20:1 and incubated at 37°C in a shaker at 180 rpm for 4 h. All experiments were repeated once and showed similar results.

To generate fully saturated Himar1 Tn mutant pools, we optimized previously described protocols step by step to test the impact of variables, including the multiplicity of infection (MOI) and transduction time and speed, as well as the bacterial density (10, 11). Transduction of *M. abscessus* at an MOI of 20:1 and incubation for 4 h in a 37°C shaker at 180 rpm yielded the highest Tn insertion frequency (Fig. 1A to C). A cell density of 8.2 × 10^10^ CFU/ml obtained by concentrating a culture at early stationary phase yielded a higher transformation frequency (9.3 × 10^−6^) than a density of 1.5 × 10^9^ CFU/ml under these conditions (Fig. 1D). A total of 10 independent Tn mutant pools were created, each containing 3.4 × 10^5^ to 1 × 10^6^ insertion events. When all 10 Tn mutant pools were combined, 7,454,000 independent insertion events were identified. Spontaneous kanamycin-resistant mutants occurred at a frequency of 1 to 3% among the Tn mutant pools, as observed in *M. avium* (11). To examine the quality of the pools, randomly selected colonies were subjected to PCR and enzyme digestion to detect the Tn and the Tn-genome junction region using primers listed in Table S1 in the supplemental material. All 40 colonies selected contained the 850-bp DNA fragment of the kanamycin resistance gene of the Himar1 Tn. Ten colonies were further analyzed and confirmed to harbor a single DNA fragment containing the junction of the Tn and a genetic element of *M. abscessus* ATCC 19977^T^, indicating a single Tn insertion event (data not shown).

10.1128/mBio.01049-21.3 TABLE S1 Primers used in this study. Download Table S1, DOCX file, 0.04 MB. Copyright © 2021 Rifat et al. 2021 Rifat et al. https://creativecommons.org/licenses/by/4.0/ This content is distributed under the terms of the Creative Commons Attribution 4.0 International license.

### Saturation and essentiality analysis of TA sites.

**FIG 2:**
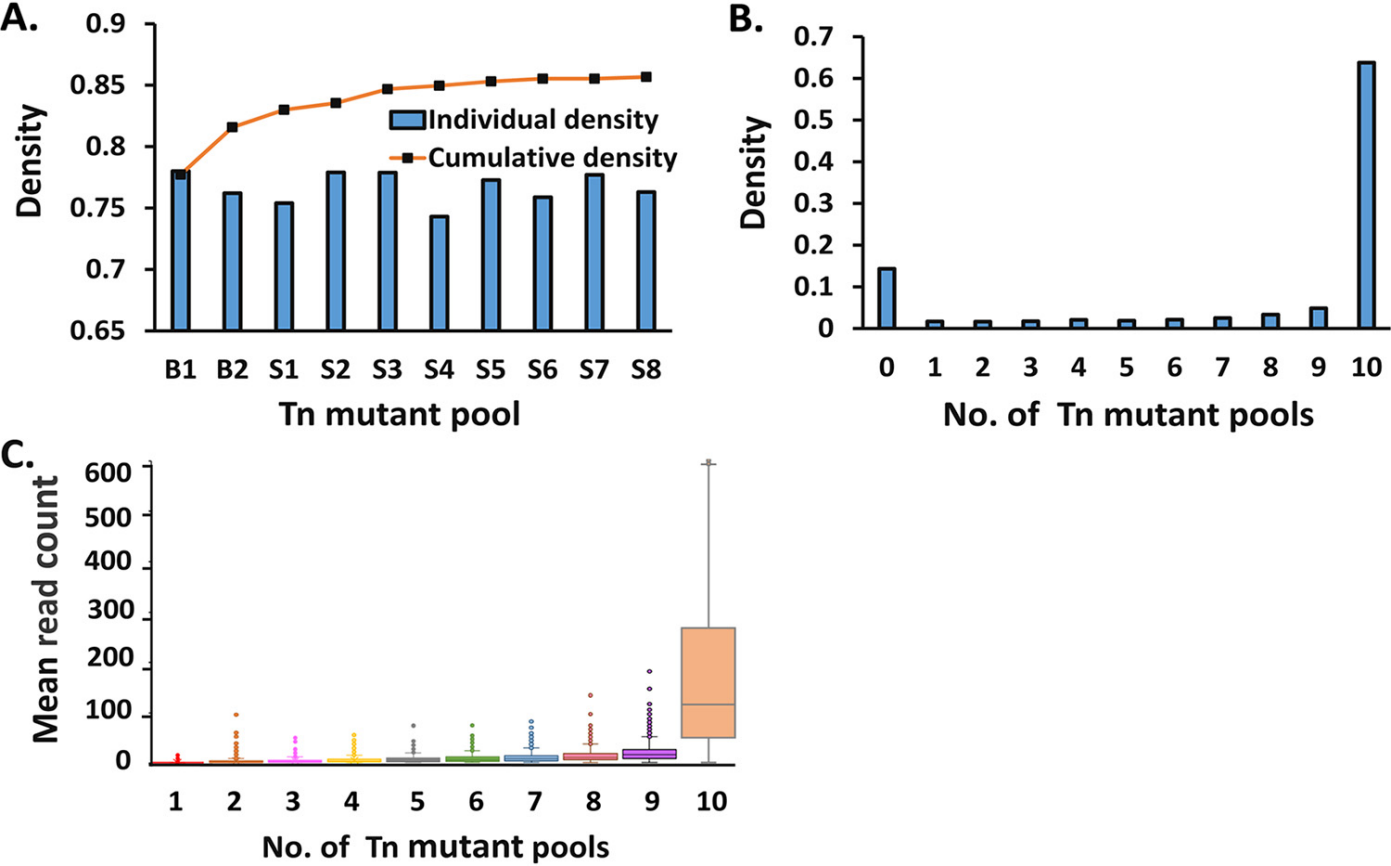
Evaluation of saturated TA sites in Tn mutant pools by deep sequencing. (A) Cumulative density of TA sites with insertions (orange line) obtained by combining independent Tn mutant pools (blue bars represent density of each individual pool). (B) Density of TA sites with insertions represented as the number of Tn mutant pools in which each particular insertion event was detected. (C) Mean read counts for TA sites with insertions detected by deep sequencing according to the number of Tn mutant pools containing that particular insertion.

To achieve full coverage of TA sites on the genome, we performed deep sequencing of Tn DNA libraries prepared in triplicate from each of the 10 independent Tn mutant pools using unique sequencing primers listed in Table S1. The resulting 30 Tn DNA libraries yielded an average of 4.5 million unique Tn-genome junctions (termed “template counts”). The average template count for each TA site is shown in Data Set S1A. Results of statistical analyses of the Tn DNA libraries after deep sequencing are summarized in Table S2. The *M. abscessus* ATCC 19977^T^ genome consists of a 5,067,172-bp circular chromosome (14) containing 91,240 TA sites. The average density of Tn insertions into TA sites for each individual Tn DNA library was 65%, but the cumulative density for each Tn pool increased to 74 to 78% after combining results from triplicate DNA libraries (see Table S2). Each pool contained 67,518 to 71,167 unique mutants. The overall insertion density achieved after cumulating the inserted TA sites identified in any of the 30 Tn DNA libraries was 85.7% (78,165 of 91,240 TA sites) (Fig. 2A). The cumulative insertion density reached a plateau after combining ≥5 of the 10 Tn mutant pools, indicating full saturation of TA sites available for insertion. Most TA sites (63.8% [58,181/91,240]) were detected in all 10 Tn pools with a mean read count of 229 per TA site, while only small proportions of inserted TA sites (i.e., 1.7 to 4.9%) with low mean read counts (i.e., 1.4 to 21) were observed in only 1 to 9 Tn pools (Fig. 2B and C). Tn insertions were not detected in the remaining 14.3% (13,075/91,240) of TA sites in any of the 10 pools (Fig. 2B), similar to results observed in *M. tuberculosis* (15.7%) and *M. avium* (16.5%) (10, 11).

10.1128/mBio.01049-21.1 DATA SET S1 Essentiality calls for the *M. abscessus* genome and plasmid pMAB23. Download Data Set S1, XLS file, 9.1 MB. Copyright © 2021 Rifat et al. 2021 Rifat et al. https://creativecommons.org/licenses/by/4.0/ This content is distributed under the terms of the Creative Commons Attribution 4.0 International license.

10.1128/mBio.01049-21.4 TABLE S2 Statistics of TA sites with Himar1 Tn insertions in 10 independent Tn mutant pools subjected to deep sequencing. Download Table S2, DOCX file, 0.06 MB. Copyright © 2021 Rifat et al. 2021 Rifat et al. https://creativecommons.org/licenses/by/4.0/ This content is distributed under the terms of the Creative Commons Attribution 4.0 International license.

The essentiality of each TA site was defined using an HMM (15, 16), a statistical model that considers read counts both at a given TA site and distributed over surrounding sites and dynamically adjusts probability distributions over states using geometric distributions to obtain locally consistent interpretations of essentiality across the genome. As such, TA sites with no insertions in nonessential regions are tolerated because neighboring sites have insertions. In contrast, if a consecutive sequence of TA sites with no insertions is long enough, the most probable state of that sequence will be assigned as essential (16). Read counts were modeled as having geometric distributions conditioned on four different states of essentiality for TA sites: essential (ES), nonessential (NE), and causing either a growth defect (GD) or growth advantage (GA) upon Tn insertion. Parameters for expected read count distributions for each state were set relative to the mean value of nonempty read counts (16). In the *M. abscessus* genome, 6.8 and 79.6% of TA sites were defined as ES and NE, respectively, for *in vitro* growth; 10.9 and 2.7% were defined as GA and GD, respectively, when disrupted (see Data Set S1A). The inferred essentiality of TA sites was used to determine the essentiality of individual coding sequences and non-ORF genomic features. The same analysis was performed on the plasmid pMAB23 harbored by *M. abscessus* ATCC 19977^T^.

**FIG 3:**
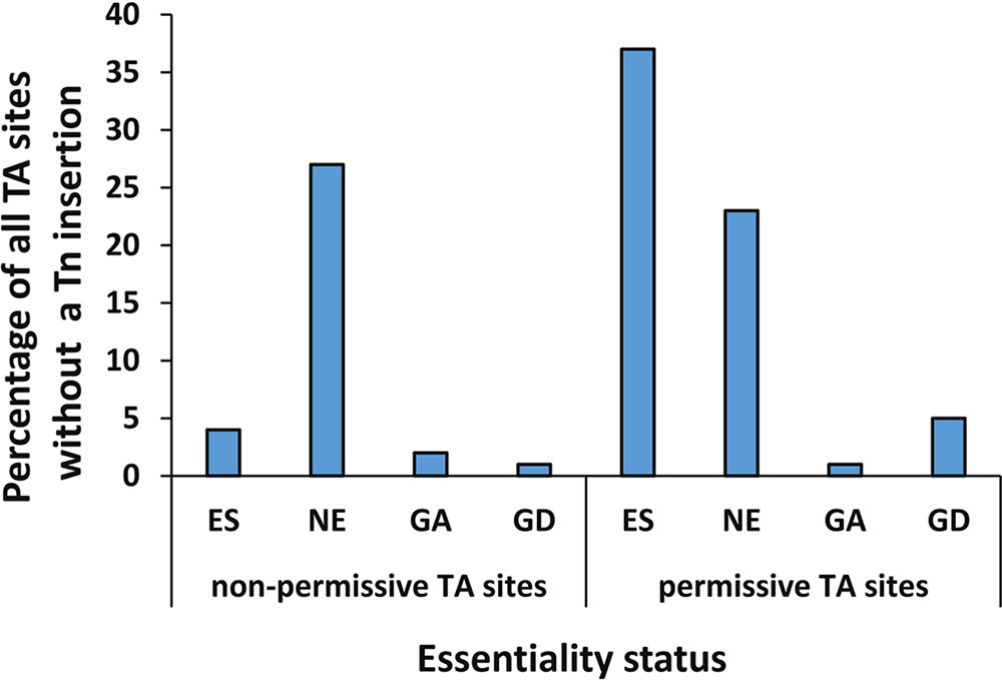
Distribution of 13,075 TA sites devoid of Tn insertions in any of 10 saturated Tn mutant pools according to the presence (4,525 TA sites) or absence (8,550 TA sites) of a previously described nonpermissive motif and the predicted essentiality status. TA sites were predicted by the four-state HMM (ES, essential; GD, growth defect when mutated; GA, growth advantage when mutated; NE, nonessential).

To better understand why some TA sites had few or no insertions or lower read counts in some number of pools (Fig. 2B and C), the TA site motif (G/C)GNTANC(G/C) identified in prior *M. tuberculosis* and *M. avium* studies as being less permissive to Himar1 insertion (10, 11) was investigated in the *M. abscessus* genome. It was greatly enriched in a set of 6,000 putative nonpermissive TA sites lacking Tn insertions compared to a set of putative permissive sites with the highest 25% of read counts. This nonpermissive motif was identified at 8.1% (7,425/91,240) of TA sites (see Data Set S1A), similar to the frequency in *M. tuberculosis* (9%) (10). Of TA sites with the nonpermissive motif, 60.9% had no Tn insertion detected in any mutant pool. Furthermore, the probability of the nonpermissive motif appearing at a TA site was inversely proportional to the number of pools in which Tn insertions were detected at that TA site (see Table S3), confirming that the nonpermissive motif was associated with lower Tn insertion frequency and lower read counts in the *M. abscessus* genome, as in *M. tuberculosis* and *M. avium* (10, 11). Remarkably, 23% of the 13,075 TA sites without a Tn insertion detected could not be explained by either a prediction of essentiality in the HMM or presence of the nonpermissive motif (Fig. 3).

10.1128/mBio.01049-21.5 TABLE S3 Distribution of TA sites with a nonpermissive motif according to the number of independent Tn mutant pools in which a Tn insertion was detected at the TA site. Download Table S3, DOCX file, 0.03 MB. Copyright © 2021 Rifat et al. 2021 Rifat et al. https://creativecommons.org/licenses/by/4.0/ This content is distributed under the terms of the Creative Commons Attribution 4.0 International license.

### Essentiality analysis of annotated genes.

Of 4920 annotated genes, 326 were identified as ES, 144 as GD, 589 as GA, and 3855 as NE (Table 1). Detailed information regarding essentiality of each gene is shown in Data Set S1B. Only six genes lacked TA loci and therefore could not be assessed by our approach. A homology comparison between proteins encoded by annotated genes of *M. abscessus* ATCC 19977^T^ (*n* = 4,920), *M. tuberculosis* H37Rv (*n* = 4,018), and *M. avium* strain MAC109 (*n* = 4,704) revealed that 44.1% (2,170/4,920) of *M. abscessus* genes shared common orthologs with both *M. tuberculosis* and *M. avium* genes, and small numbers of genes were only homologous to *M. tuberculosis* (2.5%, 122/4,920) or *M. avium* (9.7%, 477/4,920); 43.6% (2,145/4,920) of *M. abscessus* genes had no significant homology to *M. tuberculosis* or *M. avium* genes.

**TABLE 1:** Summary of essentiality analysis of *M. abscessus* ATCC 19977^T^ genome by Tn-Seq^*a*^

Genomic feature	Total no.	No. of genomic feature by assigned essentiality status				
		ES	GD	GA	NE	NA (without TA site)
ORF	4,920	326	144	589	3,855	6
sORF	126	5	0	15	89	17
ncRNA	36	4	2	5	19	6
tRNA	47	10	0	4	31	2
rRNA	3	3	0	0	0	0
Rho-independent terminator	750	20	5	46	359	320
5′ UTR	1,503	35	26	194	991	257
Promoter region	3,374	83	46	557	2,671	17

a ES, essential; GD, growth defect when mutated; GA, growth advantage when mutated; NE, nonessential; NA, not assessable using our approach due to genomic features without any TA site.

### Most essential *M. abscessus* genes are orthologs of *M. tuberculosis* genes required for *in vitro* growth.

**FIG 4:**
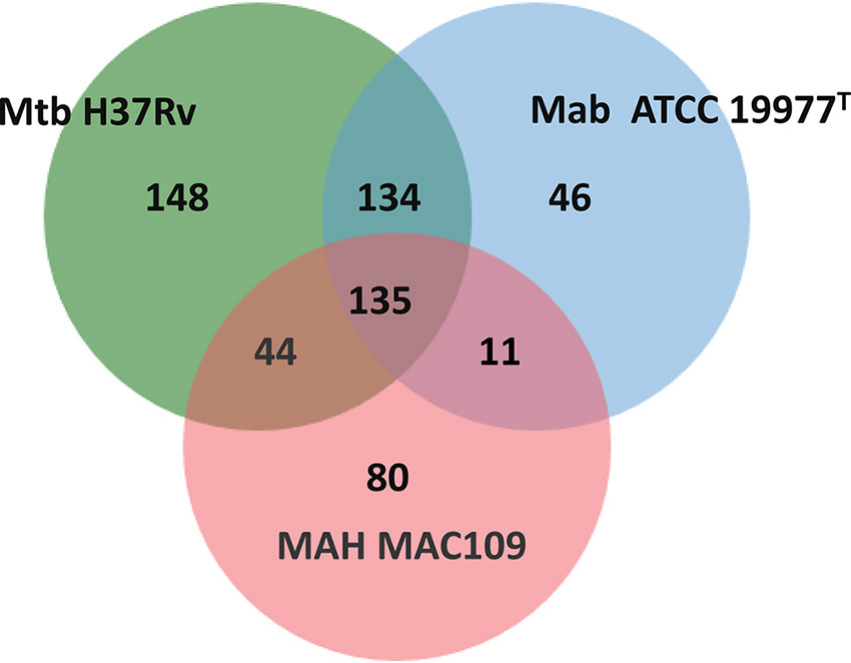
Homology comparison of essential genes among *M. abscessus* (Mab) ATCC 19977^T^, *M. tuberculosis* (Mtb) H37Rv, and *M. avium* subsp. *hominissuis* (MAH) MAC109.

A homology comparison of 326 *M. abscessus* essential genes with 461 and 270 essential genes from *M. tuberculosis* and *M. avium*, respectively (10, 11), is shown in Fig. 4. A total of 41.4% (135/326) of *M. abscessus* essential genes share mutual homology with *M. tuberculosis* and *M. avium* (see Table S4), and 41.1% have essential orthologs only in *M. tuberculosis* (see Table S5), while 3.4% (11/326) have essential orthologs only in *M. avium* (see Table S5). Interestingly, 12% (39/326) of *M. abscessus* essential genes are homologous to genes that are not essential in *M. tuberculosis* (37 genes) or *M. avium* (2 genes) (Table 2). For example, *MAB_3090c* encoding dihydrofolate reductase was defined as essential in *M. abscessus*, but its *M. tuberculosis* ortholog *Rv2763c* is not essential (10). Moreover, 2.1% (7/326) of *M. abscessus* essential genes have no homology with *M. tuberculosis* or *M. avium* genes (Table 3).

**TABLE 2:** Essential *M. abscessus* genes homologous to nonessential genes in *M. tuberculosis* H37Rv or *M. avium* MAC109

Gene^*a*^	Description
MAB_0037c	Possible serine/threonine phosphatase Ppp
MAB_0343	Aspartate kinase
MAB_0344	Aspartate-semialdehyde dehydrogenase Asd
MAB_0487	Probable cold shock protein A CspA
MAB_1513	Putative holo-[acyl-carrier-protein] synthase
MAB_1607	Possible ribonuclease E Rne
MAB_1672	GTP-binding protein Era homolog
MAB_2005	Putative cell division protein FtsW
MAB_2096c	Putative MutT/NUDIX-like protein (homologous to *M. avium* gene)
MAB_2116	Cysteinyl-tRNA synthetase CysS
MAB_2159	Conserved hypothetical protein (RNA methyltransferase?)
MAB_2778c	Probable phosphoglycerate kinase PGK
MAB_2779c	Glyceraldehyde-3-phosphate dehydrogenase, type I
MAB_2848c	Probable shikimate-5-dehydrogenase AroE
MAB_2879c	Probable protein-export membrane protein SecF
MAB_3090c	Dihydrofolate reductase DfrA
MAB_3110	Probable iron dependent transcriptional repressor FeoA
MAB_3167c	Putative penicillin-binding protein
MAB_3259c	Phosphopantetheine adenylyltransferase CoaD
MAB_3342c	Glutamyl-tRNA(Gln) amidotransferase subunit C GatC
MAB_3772c	30S ribosomal protein S11
MAB_3793c	50S ribosomal protein L15
MAB_3794c	50S ribosomal protein L30
MAB_3804c	30S ribosomal protein S14P/S29E
MAB_3812c	50S ribosomal protein L29
MAB_3991c	Possible uroporphyrin-III C-methyltransferase
MAB_4145	Probable α,α-trehalose-phosphate synthase
MAB_4953c	Membrane protein OxaA
MAB_4954c	Ribonuclease P protein component
MAB_4955c	50S ribosomal protein L34

a Genes for hypothetical proteins: MAB_1062, MAB_1669, MAB_2403, MAB_2404, MAB_2751, MAB_2893c, MAB_4077, MAB_4318 (homologous to a *M. avium* gene), and MAB_4471.

**TABLE 3:** Essential *M. abscessus* genes with no homology to *M. tuberculosis* H37Rv or *M. avium* MAC109

Gene^*a*^	Description	Phage-like element coordinates
MAB_0222c	Putative DNA-binding protein	4909957–4959626
MAB_3419	NH_3_-dependent NAD^+^ synthetase NadE	
MAB_4828c	Hypothetical protein	233621–247981

a Genes for hypothetical proteins: MAB_0210, MAB_1556, MAB_2350c, and MAB_3624c.

10.1128/mBio.01049-21.6 TABLE S4 Essential *M. abscessus* genes having homology with essential genes of both *M. tuberculosis* H37Rv and MAH MAC109. Download Table S4, DOCX file, 0.04 MB. Copyright © 2021 Rifat et al. 2021 Rifat et al. https://creativecommons.org/licenses/by/4.0/ This content is distributed under the terms of the Creative Commons Attribution 4.0 International license.

10.1128/mBio.01049-21.7 TABLE S5 Essential *M. abscessus* genes having homology with essential genes in either *M. tuberculosis* H37Rv or MAH MAC109, but not both. Download Table S5, DOCX file, 0.03 MB. Copyright © 2021 Rifat et al. 2021 Rifat et al. https://creativecommons.org/licenses/by/4.0/ This content is distributed under the terms of the Creative Commons Attribution 4.0 International license.

10.1128/mBio.01049-21.8 TABLE S6 Essentiality analysis of *M. abscessus* genes having homology with *M. tuberculosis* genes involved in type VII secretion (T7S) systems. Download Table S6, DOCX file, 0.04 MB. Copyright © 2021 Rifat et al. 2021 Rifat et al. https://creativecommons.org/licenses/by/4.0/ This content is distributed under the terms of the Creative Commons Attribution 4.0 International license.

A large number of essential genes are involved in DNA replication, RNA transcription and translation, protein folding, cell wall organization and regulation of cell shape. For example, *MAB_3869c* encodes the ortholog of the DNA-directed RNA polymerase beta chain RpoB in *M. tuberculosis* (see Table S4), the target of first-line antituberculosis (anti-TB) rifamycin drugs (17). However, this class has limited utility for treatment of *M. abscessus* infections due to intrinsic resistance (18, 19). Another large group of essential genes is associated with biosynthesis and transport of nucleotides, amino acids, fatty acids and cell wall components. All 19 genes encoding tRNA synthetases for transfer of 20 common amino acids are essential (Table 2 and Table S4). *M. abscessus* genes responsible for energy support, including *MAB_1448*-*MAB_1453* encoding the ATP synthase operon are also essential, including *atpE* (*MAB_1448*), the target of bedaquiline (see Tables S4 and S5), which potently inhibits ATP generation in *M. tuberculosis* (20) and in *M. avium* and *M. abscessus* (21–24).

### Essentiality analysis of genes involved in pathogenesis.

Functional analysis and homology comparisons identified 49 *M. abscessus* genes potentially involved in *M. abscessus* virulence, some of which were referenced from a previous study by Ripoll et al. (14) (Table 4). Of these, only four are essential for *in vitro* growth. *MAB_1933c* encodes glutamine synthetase, type I (GlnA1) (Table 4), which catalyzes ATP-dependent assimilation of ammonia into glutamate to form glutamine in *M. tuberculosis* (25). This process accounts for 15% of total ATP consumption in Escherichia coli (26, 27). *MAB_1077* encodes the two-component sensor kinase MprB (Table 4). In *M. tuberculosis*, MprAB, encoded by *Rv0981* and *Rv0982*, interacts with chaperone protein DnaK (Hsp70) to properly maintain protein homeostasis in the extracytoplasmic compartment in response to cell envelope stress (28). The other two essential genes, *MAB_0118c* and *MAB_3473c* (Table 4), encode a probable Mn-dependent superoxide dismutase and an SsrA-binding protein, respectively. The former secreted protein protects mycobacteria from reactive oxygen species, especially in macrophages (29–31). The latter is responsible for recycling stalled ribosomes and tagging incomplete nascent proteins for degradation during *trans*-translation (32, 33). *M. abscessus* only contains two type VII secretion (T7S) systems, including ESX-3 and ESX-4 loci, whereas *M. tuberculosis* harbors five T7S systems (14, 34). The essentiality and the homology comparisons for the ESX-3 and ESX-4 genes are shown in Table S6. The *M. abscessus* genes in these loci were defined as nonessential under our test conditions while, in *M. tuberculosis*, genes encoding ESX-3 were essential and those encoding ESX-4 were nonessential. The *M. abscessus* genes were homologous to *M. tuberculosis* genes except that nonessential *MAB_3760* encoding EccE4 is not found in most other interrogated mycobacteria, with the exception of M. chelonae and Mycobacterium immunogenum, thereby potentially distinguishing the *M. abscessus* ESX-4 system from that of most other mycobacteria (35, 36).

**TABLE 4:** Essentiality analysis and homology comparison of *M. abscessus* genes possibly involved in pathogenesis^*a*^

Homology search	Gene	Description	Call
Homology with *M. tuberculosis*	MAB_0942	Putative transcriptional regulator, AraC family	NE
	MAB_0945	Putative drug resistance transporter, EmrB/QacA family	NE
	MAB_3997c	Probable transcriptional regulatory protein	NE
Homology with *M. tuberculosis* and *M. avium*	MAB_0046	Probable PE family protein	NE
	MAB_0115c	Hypothetical protein	GA
	MAB_0118c	Probable superoxide dismutase [Mn]	ES
	MAB_0169c	Putative exported repetitive protein precursor	GA
	MAB_0176	Antigen 85A precursor	NE
	MAB_0283c	Hypothetical protein	NE
	MAB_0474	Hypothetical protein	NE
	MAB_0545	Protein lsr2 precursor	NE
	MAB_0580	Probable acyl-CoA dehydrogenase FadE	NE
	MAB_0593c	Probable acyl-CoA dehydrogenase FadE	NE
	MAB_0594c	Probable acyl-CoA dehydrogenase FadE	NE
	MAB_0613	Putative cytochrome P450	NE
	MAB_0615	Putative acyl-CoA dehydrogenase	NE
	MAB_0673	Putative DNA-binding response regulator PhoP	NE
	MAB_0869c	Probable resuscitation-promoting factor RpfA	NE
	MAB_0885c	Hypothetical lipoprotein LpqH precursor	NE
	MAB_0956c	Probable transcriptional regulatory protein PrrA	NE
	MAB_1069c	Probable enoyl-CoA hydratase/isomerase	NE
	MAB_1077	Probable two-component sensor kinase MprB	ES
	MAB_1918	Conserved hypothetical protein (possible hydrolase)	GA
	MAB_1933c	Probable glutamine synthetase, type I GlnA1	ES
	MAB_1945c	2-Oxoglutarate dehydrogenase, E2 component, dihydrolipoamide succinyltransferase	GD
	MAB_2124	Putative phenyloxazoline synthase MbtB	NE
	MAB_2140	NADH-quinone oxidoreductase, G subunit NuoG	NE
	MAB_2231c	Hypothetical PE family protein	NE
	MAB_2262c	Hypothetical ABC transporter ATP-binding protein	NE
	MAB_2379	Hypothetical lipoprotein LpqH precursor	NE
	MAB_2470c	Catalase/peroxidase KatG	GA
	MAB_2728c	Hypothetical invasion protein Inv1	NE
	MAB_3029	Iron-dependent repressor IdeR	GA
	MAB_3428c	Probable RNA polymerase sigma-C factor	NE
	MAB_3473c	SsrA-binding protein	ES
	MAB_3726	Putative transcriptional regulator, WhiB family	GA
	MAB_3891c	Probable transcriptional regulator, LuxR family	NE
	MAB_4083c	Heparin-binding hemagglutinin (adhesin)	GA
	MAB_4095c	Isocitrate lyase AceA	NE
	MAB_4149c	Hypothetical MCE-family protein LprN	NE
	MAB_4158	Probable acyl-CoA dehydrogenase FadE	NE
	MAB_4159	Probable acyl-CoA dehydrogenase	NE
	MAB_4184c	Superoxide dismutase [Cu-Zn] precursor	NE
	MAB_4251	Conserved hypothetical protein (peptidase?)	NE
	MAB_4336	Probable acyl-CoA dehydrogenase FadE	NE
	MAB_4402	Heat shock protein Hsp20	NE
	MAB_4407c	Putative alkylhydroperoxidase AhpD	NE
	MAB_4408c	Putative alkylhydroperoxidase AhpC	NE
Homology with *M. avium*	MAB_3123	Putative acyl-CoA dehydrogenase	NE

a CoA, coenzyme A; ES, essential; GD, growth defect when mutated; GA, growth advantage when mutated; NE, nonessential.

### Essentiality analysis of genes involved in responses to antibiotics.

Table 5 lists 58 *M. abscessus* genes possibly associated with antibiotic responses (including drug targets, drug-modifying enzymes, drug transporters, and TetR family regulators), identified through the result of comparative genomic and functional analysis. Only 16 genes were classified as essential, virtually all of them being drug targets in *M. tuberculosis* that have been validated *in vivo*. Among them, *MAB_0019* and *MAB_0006* encode DNA gyrase subunits A and B, respectively (Table 5), comprising the sole DNA topoisomerase responsible for DNA decatenation, relaxation, and supercoiling. GyrA is the target of fluoroquinolones such as moxifloxacin in TB treatment (37). The *in vivo* efficacy of moxifloxacin against *M. abscessus* remains uncertain despite it showing activity *in vitro* (38, 39). The clinical candidate SPR720 targets GyrB and is being developed for both *M. tuberculosis* and *M. abscessus* infections (40–42). *MAB_2722c* encodes the NADH-dependent enoyl-(acyl-carrier-protein) reductase that catalyzes the last step in the fatty acid elongation cycle for mycolic acid biosynthesis and *MAB_0185c* encodes arabinosyltransferase B that is necessary for arabinogalactan biosynthesis (Table 5). These enzymes are targets of the first-line anti-TB drugs isoniazid and ethambutol, respectively (43, 44), which are not recommended for treating *M. abscessus* infections due to intrinsic resistance (45). Among all MmpL family members, only *MAB_4508* encoding the trehalose monomycolate flippase MmpL3 is essential in *M. abscessus* (46) (Table 5). The β-lactamase Bla*_Mab_* encoded by *MAB_2875* is a major cause of the limited efficacy of β-lactams against *M. abscessus* (47), although a homology search identified 18 nonessential genes encoding possible β-lactamases worthy of investigation, especially those having no homology with either *M. tuberculosis* or *M. avium* (see Table S7).

**TABLE 5:** Essentiality analysis and homology comparison of *M. abscessus* genes possibly involved in responses to existing antibiotics^*a*^

Homology	Gene	Description	Call
No homology	MAB_0856c	Putative transcriptional regulator, TetR family	NE
	MAB_0591	Probable rifampin ADP-ribosyl transferase	NE
	MAB_1430c	Putative transcriptional regulator, TetR family	NE
	MAB_1496c	Putative FAD-binding monooxygenase	NE
	MAB_1497c	Putative regulatory protein, TetR family	NE
	MAB_2380c	Putative transcriptional regulator, TetR family	NE
	MAB_2385	Probable streptomycin phosphotransferase	NE
	MAB_2685	Putative transcriptional regulator, TetR family	GA
	MAB_2989	Probable chloramphenicol acetyltransferase	NE
	MAB_4320c	Putative transcriptional regulator, TetR family	NE
	MAB_4384	TetR regulator	NE
	MAB_4532c	Gcn5-related *N*-acetyltransferase (GNAT) Eis2	NE
Homology with *M. tuberculosis*	MAB_0163c	Probable phosphotransferase	NE
	MAB_0945	Putative drug resistance transporter, EmrB/QacA family	NE
	MAB_1448	ATP synthase C chain AtpE	ES
	MAB_1858	Probable ABC transporter antibiotic-transport ATP-binding protein	NE
	MAB_1859	Probable ABC transporter antibiotic-transport integral membrane protein	NE
	MAB_1860	Probable ABC transporter antibiotic-transport integral membrane protein	NE
	MAB_2297	23S rRNA (adenine(2058)-N(6))-methyltransferase Erm(41)	NE
	MAB_2875	β-Lactamase precursor (Penicillinase)	NE
	MAB_3080c	Dihydrofolate reductase DfrA	ES
	MAB_4283c	Conserved hypothetical protein (isoniazid-inducible gene protein IniA?)	NE
	MAB_4395	Aminoglycoside 2′-N-acetyltransferase	NE
Homology with *M. tuberculosis* and *M. avium*	MAB_0006	DNA gyrase (subunit B) GyrB (DNA topoisomerase)	ES
	MAB_0019	DNA gyrase (subunit A) GyrA (DNA topoisomerase)	ES
	MAB_0173	Prenyltransferase family protein UbiA	ES
	MAB_0180	Polyketide synthase PKS13	ES
	MAB_0185c	Probable arabinosyltransferase B	ES
	MAB_0189c	Probable arabinosyltransferase C	GD
	MAB_0192c	Probable oxidoreductase	ES
	MAB_0408c	Probable bifunctional membrane-associated penicillin-binding protein PonA2/glycosyl transferase	GA
	MAB_1134c	Probable membrane protein MmpL	NE
	MAB_2301	Probable membrane protein MmpL	GA
	MAB_1359c	Putative ABC transporter, ATP-binding protein	NE
	MAB_1409c	Multidrug efflux transporter Tap	NE
	MAB_1472c	Putative nicotinamidase/pyrazinamidase	NE
	MAB_1560	Probable ABC transporter (macrolide-transport) ATP-binding protein	ES
	MAB_1689	Probable daunorubicin resistance ABC transporter ATP-binding subunit DrrA	NE
	MAB_1877c	3-Oxoacyl-[acyl-carrier-protein] synthase 1 KasA	ES
	MAB_2108	Probable undecaprenyl-diphosphatase (Bacitracin resistance protein)	NE
	MAB_2208c	Hypothetical protein	GA
	MAB_2319c	Probable lysyl-tRNA synthetase 2 LysX	NE
	MAB_2415c	Conserved hypothetical protein (penicillinase repressor?)	GA
	MAB_2643c	Tryptophan synthase, alpha subunit TrpA	ES
	MAB_2644c	Tryptophan synthase, beta subunit TrpB	ES
	MAB_2705c	Isoleucyl-tRNA synthetase IleS	ES
	MAB_2722c	Enoyl-(acyl-carrier-protein) reductase (NADH)	ES
	MAB_3508c	Putative transcriptional regulator	NA
	MAB_2299c	TetR regulator	GA
	MAB_4382c	Putative membrane protein MmpL5	NE
	MAB_4383c	Putative membrane protein MmpS5	NE
	MAB_4482	Putative phosphotransferase	NE
	MAB_4659	Conserved hypothetical protein (phosphoribosyltransferase?)	NE
	MAB_4923	Leucyl-tRNA synthetase	ES
Homology with *M. avium*	MAB_1135c	Probable membrane protein MmpS	GA
	MAB_2300	Probable membrane protein MmpS	GA
	MAB_3449c	Putative transporter	NE
	MAB_4508	Putative membrane protein MmpL	ES

a ES, essential; GD, growth defect when mutated; GA, growth advantage when mutated; NE, nonessential.

10.1128/mBio.01049-21.9 TABLE S7 Essentiality analysis and homology comparison of *M. abscessus* genes with homology to β-lactamases. Download Table S7, DOCX file, 0.03 MB. Copyright © 2021 Rifat et al. 2021 Rifat et al. https://creativecommons.org/licenses/by/4.0/ This content is distributed under the terms of the Creative Commons Attribution 4.0 International license.

### Essentiality analysis of genes involved in peptidoglycan metabolism.

Mycolyl-arabinogalactan-peptidoglycan complex is the key structure for the mycobacterial cell wall that contributes to the intrinsic resistance to a number of antibiotics. Consequently, it is a major target for drug development (48). Table 6 lists genes associated with peptidoglycan (PG) biosynthesis and remodeling, as previously identified and categorized (49–51). The essentiality comparisons of these 28 *M. abscessus* genes with 30 genes in *M. tuberculosis* H37Rv (10) indicated a few differences. For example, *Rv0024* and *rpfD* orthologs are absent in *M. abscessus* and *MAB_3167c* encoding a putative penicillin-binding protein is essential in *M. abscessus*, but its ortholog Rv2864c is nonessential in *M. tuberculosis* (Table 6). Similarly, *ripA* encoding an endopeptidase contains an essential domain in *M. tuberculosis*, but its ortholog *MAB_2728c* was classified as nonessential (Table 6). Remarkably, 11 *M. abscessus* genes were classified as GA genes compared to only one in *M. tuberculosis* (Table 6). The GA *M. abscessus* genes were found in each functional group, and their corresponding orthologs in *M. tuberculosis* are well characterized regarding their roles in PG biosynthesis (52–59). Among them, *ldt_Mab1_* and *ldt_Mab2_* were confirmed to encode l,d-transpeptidases in *M. abscessus* like their orthologs *ldt_Mt1_* and *ldt_Mt2_*, which are targets of cephalosporin and carbapenem β-lactams (55).

**TABLE 6:** Essentiality analysis and homology comparison of genes associated with peptidoglycan biosynthesis and remodeling in *M. abscessus* ATCC 19977^T^ and *M. tuberculosis* H37Rv^*a*^

Category	*M. tuberculosis* gene	Call	*M. abscessus* gene	Call	Description of *M. abscessus* gene
Resuscitation promoting factors	*rpfA* (Rv0867c)	NE	MAB_0869c	NE	Probable resuscitation-promoting factor RpfA
	*rpfB* (Rv1009)	NE	MAB_1130	NE	Hypothetical protein
	*rpfC* (Rv1884c)	NE	MAB_4080c	NE	Hypothetical protein
	*rpfD* (Rv2389c)	NE	NA		NA
	*rpfE* (Rv2450)	NE	MAB_1597	GA	Hypothetical protein
Penicillin-binding proteins and noncanonical transglycosylases	*ponA1* (Rv0050)	GA	MAB_4901c	GA	Penicillin-binding protein
	*ponA2* (Rv3682)	NE	MAB_0408c	GA	Probable bifunctional membrane-associated penicillin-binding protein PonA2/glycosyl transferase
	*pbpA* (Rv0016c)	NE	MAB_0035c	GA	Probable penicillin-binding protein PbpA
	*pbpB* (Rv2163c)	ESD	MAB_2000	ES	Probable penicillin-binding membrane protein PbpB
	Rv2864c	NE	MAB_3167c	ES	Putative penicillin-binding lipoprotein
	*dacB1* (Rv3330)	NE	MAB_3681	NE	Probable penicillin-binding protein DacB1
	*dacB2* (Rv2911)	NE	MAB_3234	GA	Probable d-alanyl-d-alanine carboxypeptidase DacB2
	Rv3627c	GD	MAB_0519	NE	Peptidase S13 (d-alanyl-d-alanine carboxypeptidase)
	*ftsW* (Rv2154)	ES	MAB_2005	ES	Putative cell division protein
	*rodA* (Rv0017)	NE	MAB_0036c	GA	Probable cell division protein
	MSMEG_1900	NE	MAB_2019	NE	Putative VanY-type carboxypeptidase
Endopeptidases	Rv0024	NE	NA		NA
	*ripA* (Rv1477)	ESD	MAB_2728	NE	Hypothetical invasion protein Inv1
	*ripB* (Rv1478)	NE	MAB_2727c	NE	Hypothetical invasion protein Inv2
	*ripD* (Rv1566c)	NE	MAB_2474	NE	Hypothetical protein
	Rv2190c	NE	MAB_1974	GA	Putative secreted protein
l,d-Transpeptidases	*ldt*_Mt1_ (Rv0116c)	NE	MAB_3165c	GA	Hypothetical protein
	*ldt*_Mt2_ (Rv2518c)	NE	MAB_1530	GA	Probable conserved lipoprotein LppS
	*ldt*_Mt3_ (Rv1433)	NE	MAB_4775c	NE	Hypothetical protein
	*ldt*_Mt4_ (Rv0192)	NE	MAB_4537c	NE	Hypothetical protein
	*ldt*_Mt5_ (Rv0482)	NE	MAB_4061c	NE	Hypothetical protein
Amidases	*ami1* (Rv3717)	NE	MAB_0318c	GA	Hypothetical protein
	*ami2* (Rv3915)	ES	MAB_4942	ES	*N*-Acetylmuramoyl-l-alanine amidase CwlM
	*ami3* (Rv3811)	NE	MAB_0168c	GA	Putative *N*-acetymuramoyl-l-alanine amidase
	*ami4* (Rv3594)	NE	MAB_4807	NE	Bacteriophage protein

a Candidate genes were sourced and classified based on previous reports (49–51). The essentiality status of *M. tuberculosis* genes was referenced from DeJesus et al. (10). ES, essential; GD, growth defect when mutated; GA, growth advantage when mutated; NE, nonessential.

### Essential *M. abscessus* genes without homologs in *M. tuberculosis* or *M. avium*.

Most of the 43.6% of *M. abscessus* genes (2,145/4,920) with no significant similarity to either *M. tuberculosis* or *M. avium* are hypothetical genes. The majority were defined as nonessential, whereas 262 and 16 genes conferred a growth advantage and a growth defect, respectively, when interrupted. Four genes without TA sites could not be assessed. Many TetR family regulators, *whiB* regulators, drug-modifying enzymes, and efflux pumps belonged to this category, some of which are known to be involved in intrinsic drug resistance, such as *MAB_0591* (rifampin ADP-ribosyl transferase) (19), *MAB_4532c* (*eis2*, Gcn5-related *N*-acetyltransferase) (60, 61), and *MAB_4384* (TetR regulator) (62). Only seven essential *M. abscessus* genes lacked significant homology with *M. tuberculosis* or *M. avium* genes, most of which encode hypothetical proteins (Table 3). However, *MAB_3419* encodes NH_3_-dependent NAD^+^ synthetase (NadE) (63), which catalyzes the last step in *de novo* NAD^+^ biosynthesis, and is a validated drug target in *M. tuberculosis* (64–66). Almost all external genes, including an 81-kb full-length prophage, 3 prophage-like elements, and 17 clusters of horizontally transferred genes from nonmycobacterial organisms described by Ripoll et al. (14), are not homologous to *M. tuberculosis* or *M. avium* genes and are dispensable for *in vitro* growth. However, two essential genes, *MAB_0222c* (putative DNA-binding protein) and *MAB_4828c* (unknown function) are located on distinct prophage-like elements (Table 3). Analysis of peptide sequences from 1,718 *M. abscessus* complex genomes deposited in GenBank using BLAST revealed homologs of *MAB_0222c* and *MAB_4828c* in only 23 and 307 genomes, respectively, and exclusively in *M. abscessus* subsp. *abscessus*. In addition, MAB_0222c showed the highest identity of 63% with a DNA-binding protein in M. chelonae, whereas MAB_4828c showed 72 and 75% identities to hypothetical proteins in *Mycobacteroides salmoniphilum* and *Mycobacteroides franklinii*, respectively. These strains were isolated from water and have caused fish and, rarely, human infections (67). Further comparative genomic analysis showed that 80% (3,940/4,920) of *M. abscessus* genes have homologs in M. chelonae type strain CCUG 47445 (68), including 96% (313/326) of essential *M. abscessus* genes (data not shown). Table S8 lists 13 essential genes without significant homologs in M. chelonae CCUG 47445. Interestingly, the MAB_0222c homolog was not identified in this strain.

10.1128/mBio.01049-21.10 TABLE S8 Essential *M. abscessus* genes that lack homologs in *M. chelonae* type strain CCUG 47445. Download Table S8, DOCX file, 0.02 MB. Copyright © 2021 Rifat et al. 2021 Rifat et al. https://creativecommons.org/licenses/by/4.0/ This content is distributed under the terms of the Creative Commons Attribution 4.0 International license.

### Essentiality analysis of short ORFs and non-ORF genomic features.

Short ORFs (sORFs) and non-ORF genomic features in *M. abscessus* ATCC 19977^T^ were first identified by Miranda-CasoLuengo et al. using RNA-seq/Ribo-seq and proteomics technologies (69). In all, 126 ribosomally protected sORFs were detected over the genome, 80% of which are ≤50 amino acids in length (69). Table 1 shows the essentiality assignments of sORFs, ncRNAs (noncoding RNAs), tRNAs (transfer RNAs), rRNAs (ribosomal RNAs), 5′ UTRs (5′ untranslated regions), promoter regions, and Rho-independent terminators. Essential genetic elements are shown in Table 7. Only 5 of 126 sORFs were defined as essential, although 17 without TA sites could not be assessed. Of 36 ncRNAs containing 55 to 404 nucleotides, 4 were classified as essential. Of 47 tRNA genes, 10 were classified as essential, while 2 without TA sites could not be assessed. Remarkably, 4 tRNA genes conferred a growth advantage when disrupted, and only 2 of 7 singleton tRNA genes (MAB_t5001 [Ile, GAT] and MAB_t5014 [Asp, GTC]) were essential. Interestingly, one or two copies of tRNA genes for transfer of Gln and Thr were essential in *M. abscessus*, *M. tuberculosis*, and *M. avium* (10, 11), suggesting that these two amino acids play crucial roles in mycobacterial physiology. As expected, the genes encoding 16S, 23S, and 5S rRNAs were essential in *M. abscessus* as in *M. tuberculosis* and *M. avium* (10, 11). 16S and 23S rRNAs are targets of amikacin and macrolides, respectively, which are first-line drugs recommended for treating *M. abscessus* infections (45). Most predicted promoter regions, 5′ UTRs and rho-independent terminators were nonessential. Detailed information on the essentiality analyses of sORFs and non-ORF genomic features is available in Data Set S1C.

**TABLE 7:** Other essential genomic features of *M. abscessus* ATCC 19977^T^

sORF	ncRNA	tRNA	rRNA
MAB_5003	ncRNA_Mab1237c	MAB_t5001 (GAT,Ile)	MAB_r5051 (16sRNA,rrs)
MAB_5017c	ncRNA_Mab13471c	MAB_t5002 (TGC,Ala)	MAB_r5052 (23sRNA,rrl)
MAB_5034c	ncRNA_Mab1913	MAB_t5013 (TTC,Glu)	MAB_r5053 (5sRNA,rrf)
MAB_5035c	ncRNA_Mabr5052	MAB_t5014 (GTC,Asp)	
MAB_5050c		MAB_t5025c (TCC,Gly)	
		MAB_t5031c (GAG,Leu)	
		MAB_t5040c (CTG,Gln)	
		MAB_t5042c (CAT,Met)	
		MAB_t5044c (CAT,Met)	
		MAB_t5045c (GGT,Thr)	

### Essentiality analysis of the plasmid.

*M. abscessus* ATCC 19977^T^ contains the 23-kb plasmid pMAB23, which is 99% identical to pMM23 from Mycobacterium marinum ATCC BAA-535 and harbors a putative mercury resistance operon (14, 70). All of the 22 annotated coding sequences and intergenic regions are dispensable for *in vitro* growth except *MAB_p16c* encoding putative replication protein RepA and the intergenic region upstream of *repA* (between *MAB_p17* and *MAB_p16c*), which were defined as GD when disrupted (see Data Set S1D).

## DISCUSSION

Despite its increasing incidence as a cause of difficult-to-treat opportunistic infections, *M. abscessus* remains poorly studied, in part because it has been difficult to manipulate genetically. The availability of a comprehensive list of essential genes and other genomic features is a crucial step toward greater understanding of M. abscessus physiology and pathogenesis. To our knowledge, this is the first comprehensive analysis of the essentiality of *M. abscessus* genetic elements required for *in vitro* growth using fully saturated Tn mutant pools and deep sequencing.

Use of highly saturated Tn mutant libraries and an HMM increases the predictive accuracy of essentiality analyses, especially for classifying genomic features with fewer TA sites (10, 11). HMMs have proven to be a reliable statistical method for estimating different degrees of essentiality across the genome in an unbiased (non-gene-centered) way (10, 11, 16). However, it has been difficult to achieve high saturation using the Himar1 Tn in *M. abscessus*. To date, published reports studied *M. abscessus* Tn mutant pools containing only 6,000 to 8,000 unique Tn mutants (9, 36), far from the 91,240 TA sites in the genome of *M. abscessus* ATCC 19977^T^. Through systematic optimization of procedures for Himar1 Tn mutagenesis and use of triplicate Tn DNA libraries per Tn mutant pool, we increased the number of unique TA insertions to 67,518 to 71,167 per pool and achieved full saturation of detectable TA site insertions. The overall proportions of TA sites with or without observed Tn insertions (85.7 and 14.3%, respectively) and with a known nonpermissive motif (8.1%) are similar to those reported in *M. tuberculosis* and *M. avium* (10, 11). Our findings also indicate that, in addition to the lethality of Tn insertions into essential genes and the restrictive effects of the nonpermissive motif (10), other unknown factors may restrict Himar1 insertion into certain TA sites. The Himar1 Tn is widely used in mutagenesis studies, but its utility and limitations in diverse mycobacterial genomes is understudied. Our optimization approach may provide a useful roadmap for generating more fully saturated Tn mutant pools in other mycobacteria.

The *M. abscessus* complex is a large and heterogeneous group of species (1, 71, 72) capable of causing opportunistic infections in any organ, but commonly in the lungs and skin and soft tissue (73). In-depth genomic analysis of *M. abscessus* indicates a nonconservative genome, in which the core genome is limited to 64.15% of the pan-genome, differing from the conservative pathogen *M. tuberculosis*, whose core genome represents 96.1% of the pan-genome (72). Despite *M. abscessus* diversity in genome size and content, our findings on the essentiality of genomic elements of *M. abscessus* ATCC 19977^T^ will shed light on other *M. abscessus* complex strains, especially many clinically relevant strains in the United States and Europe, since phylogenomic analyses place this type strain within the predominant clone observed in several global and national studies of clinical isolates (74). Most essential *M. abscessus* genes defined here are highly homologous to those identified in similar studies of *M. tuberculosis* and *M. avium*. These results provide a fundamental basis for utilizing available knowledge and approaches from *M. tuberculosis* and *M. avium* studies to promote research to address key knowledge gaps regarding *M. abscessus*. Our findings also highlight intriguing genomic differences that could be exploited for greater understanding of *M. abscessus* pathogenesis and development of new tools to treat and prevent *M. abscessus* infections.

Essential *M. abscessus* genes sharing significant homology with essential *M. tuberculosis* genes include validated targets for important anti-TB drugs, such as isoniazid (43), rifampin (17), ethambutol (44), moxifloxacin (37), and bedaquiline (20). However, these drugs are not effective against *M. abscessus* infections or, in the case of bedaquiline, require further study (21, 22, 38, 45). Thus, drugs developed and optimized against essential *M. tuberculosis* targets may not be useful against even highly homologous essential targets in *M. abscessus* due to interspecies differences in target protein structure or the presence or absence of enzymes that activate prodrugs like isoniazid or inactivate drugs, such as rifamycins, or other unique resistance mechanisms, such as efflux transporters (19, 47, 60–62, 75–78). Thus, developing new anti-*M. abscessus* drugs against drug targets validated in TB should be an effective approach, but programs focused specifically on *M. abscessus* are needed to deliver optimized drugs that exploit interspecies differences in structure-activity relationships (SAR) and intrinsic resistance mechanisms. For example, our approach predicted MmpL3 (MAB_4508) to be essential in *M. abscessus*, as in *M. tuberculosis*. This flippase required for translocating mycolate precursors to the cell envelope was successfully targeted first in *M. tuberculosis* by a series of indole-2-carboxamide inhibitors but subsequent evolution of this series and others based on unique SAR delivered compounds with superior *in vitro* and *in vivo* activity against *M. abscessus* (46, 79–82). Glutamine synthase GlnA1 (MAB_1933c) is predicted to be essential in *M. abscessus* and may represent a more novel drug target and virulence factor. The attenuation of an *M. tuberculosis glnA1* deletion mutant during glutamine auxotrophy and in guinea pigs and mice is encouraging in this regard (83, 84), especially since glutamine is not readily available in CF sputum, an important niche for *M. abscessus* (85). Furthermore, genetic or chemical disruption of GlnA1 increases vulnerability to bedaquiline in *M. tuberculosis* (27), suggesting that a MAB_1933c inhibitor could synergize with diarylquinolines against *M. abscessus*.

Genes essential in *M. abscessus* but not in *M. tuberculosis* could also be more effectively exploited as drug targets in *M. abscessus*. *MAB_3090c* encodes the dihydrofolate reductase (DHFR) DfrA, a conserved enzyme in the folate biosynthesis pathway (86). Although DHFR inhibitors are effective anti-proliferative drug targets for treating a variety of malignancies and autoimmune and infectious diseases (86, 87), they have not proven very effective against *M. tuberculosis* (88). However, DHFR is only conditionally essential in *M. tuberculosis* (10) and is not particularly vulnerable because its loss can be compensated by upregulation of a second DHFR enzyme, Rv2671, and ThyX (89, 90). Whether the nonessential Rv2671 ortholog MAB_2976 and ThyX (Rv2754c) ortholog MAB_3085c that showed a growth advantage after disruption can compensate for the loss of DfrA in *M. abscessus* may determine the value of this target in *M. abscessus*.

Genes essential in *M. abscessus* that have limited or no homology with genes in *M. tuberculosis* may represent new and more specific drug targets. Of particular interest is *MAB_3419*, a putative ammonia-dependent NAD synthetase (NadE) that catalyzes the final step in NAD^+^ biosynthesis. NAD^+^ is an essential cofactor that mycobacteria synthesize either *de novo* from aspartate or from nicotinamide/nicotinic acid scavenged from the environment. Both pathways utilize NadE. Interestingly, *MAB_3419* is predicted to be a single-domain NAD^+^ synthetase that uses ammonia as a nitrogen source, with >90% protein sequence homology with NadE in Mycobacterium chelonae and a few other rapidly growing mycobacteria but limited homology with the *M. tuberculosis* and Mycobacterium smegmatis enzymes, which are glutamine-dependent NAD+ synthetases comprised of a C-terminal NAD^+^ synthetase domain fused with an N-terminal glutaminase domain. NadE is a genetically and chemically validated drug target in *M. tuberculosis* l (91–93), but the described inhibitors bind to sites not present in MAB_3419, indicating a different chemical route is required to target *M. abscessus* NadE.

The mycobacterial cell wall is an essential structure for growth and virulence. Comprised of three distinct layers (PG, arabinogalactan, and mycolic acids), it is an attractive target for antimycobacterial antibiotics (48). Unlike in TB, PG synthesis inhibitors, i.e., imipenem and cefoxitin are already first-line drugs for *M. abscessus* infections. PG requires constant expansion, remodeling and recycling during bacterial growth and division (94). We identified interesting differences in the essentiality of genes associated with PG metabolism between *M. abscessus* and *M. tuberculosis*. Although the growth-advantaged phenotypes of Tn insertions in 11 PG-associated *M. abscessus* genes need to be confirmed, we speculate that *M. abscessus* has evolved interaction networks that differ from those in *M. tuberculosis* and may confer a greater ability to compensate for disruption of certain PG-synthesizing enzymes to ensure cell wall integrity and greater adaptability to changing environmental conditions.

Approximately 5 to 6% of genes in the *M. abscessus* genome were likely acquired through horizontal gene transfer from other organisms (14). Among them, only two genes from prophage-like elements (*MAB_0222c* and *MAB_4828c*) are defined as essential for *in vitro* growth. Horizontal gene transfer preferentially occurs between specific groups of organisms that share ancestry or habitat, presumably under evolutionary pressure (95, 96). The genes’ homology to M. chelonae and to *M. salmoniphilum* and *M. franklinii*, respectively, suggests horizontal gene transfer in a shared habitat, perhaps in water (67). Since the genes are found in the more pathogenic *M. abscessus* subsp. *abscessus* but not in *M. abscessus* subsp. *massiliense* or *M. abscessus* subsp. *bolletii*, one wonders whether their acquisition improves pathogenicity in addition to adaptation and survival in the environment.

We evaluated the essentiality of 126 previously identified sORFs (69). Interestingly, essential sORFs also showed significant responses to stress conditions in previous RNA-seq studies. For instance, *MAB_5003*, *MAB_5034c*, and *MAB_5035c* are significantly upregulated, while *MAB_5050c* is downregulated in artificial sputum, and increased expression of *MAB_5035c* was detected in response to kanamycin (69). *MAB_5050c* is located at the leader region of essential gene *MAB_3798c* encoding 30S ribosomal protein S8 (RpsH) that directly binds to 16S rRNA to assemble the 30S subunit of the ribosome for protein synthesis (97). RpsH also plays a critical role in selectively inhibiting synthesis of ribosomal proteins whose genes are in the same *spc* operon as its own in response to changing environmental conditions (98). Although any functional association between MAB_5050c and RpsH requires further investigation, downregulation of MAB_5050c may play some role in regulating protein synthesis under stress. tRNAs play a central role in protein translation and thus are potential targets for new antibiotics that inhibit attachment of amino acids onto corresponding tRNAs. Most *M. abscessus* tRNA genes are dispensable for *in vitro* growth, as in *M. tuberculosis* and *M. avium* (10, 11). Only two of seven singleton tRNAs were defined as essential, similar to findings in Saccharomyces cerevisiae, in which four of six singleton tRNA genes are essential and most tRNA deletions do not cause altered growth phenotypes in rich medium (99), presumably because they are compensated by members of the same or different anti-codon families in many conditions (99).

We defined essential genes under optimal *in vitro* growth conditions using a reliable and comprehensive approach. However, further confirmation of essentiality may still be necessary. Genes not required for *in vitro* growth could become conditionally essential when *M. abscessus* encounters certain stresses during infection, including in the nutritionally aberrant environments of cystic fibrosis airway mucus, biofilms, in phagocytes, and necrotic pyogranulomatous lesions. For example, although genes comprising the ESX-3 and ESX-4 loci are nonessential under conditions in this study, *M. abscessus* ESX-3 plays an important role in pathogenesis (100), and *M. abscessus* ESX-4 genes are required for intracellular survival (36). Nevertheless, differences in essentiality and homology between these genes in *M. abscessus* and *M. tuberculosis* may indicate differences in function, which deserves further study. Using saturated *M. abscessus* Tn mutant libraries to identify new virulence factors and potential drug targets under clinically relevant conditions is under investigation.

## MATERIALS AND METHODS

### Bacterial strain, bacteriophage, and media.

*M. abscessus* ATCC 19977^T^ type strain was purchased from the American Type Culture Collection (ATCC). Mycobacterium smegmatis mc^2^155 and bacteriophage ΦmycomarT7 came from stocks described previously (101). Unless stated otherwise, Middlebrook 7H9 broth base (Difco, BD) supplemented with 10% oleic acid-albumin-dextrose-catalase (OADC) complex (BD), 0.5% glycerol, and 0.05% Tween 80 (Sigma-Aldrich) (7H9 broth) were used for cultivation. 7H11 agar (Difco, BD) containing 10% OADC, 0.5% glycerol, and 0.1% Tween 80 were used to select Tn mutants (7H11 agar). Top agar containing 0.5 g of 7H9 broth base, 0.7 g of Bacto agar (Difco, BD), and 0.5 ml of glycerol in 100 ml of distilled water was prepared and poured on 7H11 agar plates when needed for phage studies.

### Construction of Himar1 Tn mutant pools.

A protocol for constructing a saturated Himar1 Tn mutant pool was carefully optimized based on the literatures (11, 102). Detailed information was provided in the supplemental material.

### Preparation of DNA libraries of Tn mutant pools.

DNA libraries of Tn mutant pools were prepared as previously described with modification (11, 102). Detailed methods are provided in Text S1 in the supplemental material.

10.1128/mBio.01049-21.2 TEXT S1 Construction of Himar1 transposon mutant pools and preparation of DNA libraries. Download Text S1, DOCX file, 0.04 MB. Copyright © 2021 Rifat et al. 2021 Rifat et al. https://creativecommons.org/licenses/by/4.0/ This content is distributed under the terms of the Creative Commons Attribution 4.0 International license.

### Deep sequencing of Tn insertions and analysis of sequencing data.

Thirty Tn mutant DNA libraries representing triplicate samples from each of 10 Tn mutant pools were sequenced on an Illumina HiSeq instrument, collecting 18 (10.4 to 31.5) million 150-bp paired-end reads per sample. The reads were filtered by Trimmomatic v0.39 (103) and processed using TPP in TRANSIT, which counts reads mapping to each TA dinucleotide site against the published *M. abscessus* ATCC 19977^T^ (accession number CU458896) genome sequence (after eliminating reads sharing the same template barcode) (15). A Hidden Markov Model (HMM) offered by TRANSIT was used to assign the most probable state of essentiality of the sequence of TA sites based on the read count at the site and the distribution over the surrounding sites (15, 16). It parses a genome into contiguous regions belonging to one of four essentiality states—essential (ES), nonessential (NE), growth defect when mutated (GD), or growth advantage when mutated (GA), based on local insertion density and mean value of nonempty read counts at TA sites (with ES being near 0, NE being near the mean, and GD and GA being approximately 1/10 and 5 times the mean, respectively). To search for TA site motifs that are less permissive for Himar1 Tn insertion in the *M. abscessus* genome, all TA sites without insertions but excluding TA sites from essential gene regions or regulatory regions which may be under selection pressure were selected as a putative nonpermissive set (∼6,000 TA sites). The TA site regions with the top 25% read counts of Himar1 Tn insertions were then chosen as a putative permissive set. The nucleotides surrounding TA sites were compared between the two sets.

Ortholog analysis was performed among all annotated genes and the subset of predicted essential genes of *M. abscessus* ATCC 19977^T^, *M. tuberculosis* H37Rv, and *M. avium* MAC109 (10, 11) using orthovenn2 diagram analysis (excluding genes encoding proteins containing less than 20 amino acids) (104). Homology searches were performed between the genomes of *M. abscessus* ATCC 19977^T^ and *M. chelonae* type strain CCUG 47445 (68) using the same method. Other genomic features, including new sORFs and non-ORF genomic elements, were identified based on published information (69). Promoter regions were defined based on a set of 2,653 transcriptional start sites (TSSs) defined previously (69). A region around each TSS (bp −150 to +70) was used to determine the promoter region as described previously (10). Lastly, Rho-independent transcription terminators were predicted using the ARNold online tool (http://rssf.i2bc.paris-saclay.fr/toolbox/arnold/) (105).

### Data availability.

The raw data are deposited in NCBI SRA database under BioSample accession numbers SAMN16825978 to SAMN16826007.
