# Supplementary material for: Genome-Wide Essentiality Analysis of Mycobacterium abscessus by Saturated Transposon Mutagenesis and Deep Sequencing
Source: mBio. 2021 Jun 15;12(3):e01049-21. doi: 10.1128/mBio.01049-21 (PMC8262987; doi:10.1128/mBio.01049-21)
Supplement: TABLE S1 [file mbio.01049-21-st001.docx]

**Table S1. List of primers used in the study**

| **Name of primer or adapter** | **Sequence of primer or adapter** |
| --- | --- |
| Kan-F | GTG AAA ATT CAC CGA GGC AG |
| Kan-R | GTA TTT CGT CTC ACT CAG GC |
| AdBTM | 5phos/ ACC AGC CCG GGC /3AmMoO(NH_2_) |
| AdTOP | GTA ATA CGA CTC ACT ATA GGG CAC GCG TGG TCG ACG GCC CGG GCT GGT |
| T7-A | TAA TAC GAC TCA CTA TAG GGT CTA GAG A |
| AP1 | GTA ATA CGA CTC ACT ATA GGG CAC |
| Adapter 1.2 | 5Phos/ TAC CAC GAC CA /3AmMO (NH_2_) |
| Adapter 2.2 Bar B | ATG ATG GCC GGT GGA TTT GTG NNA NNA NNN TGG TCG TGG TAT |
| JEL_API | ATG ATG GCC GGT GGA TTT GTG |
| Reverse sequencing primer Sol_Mar mixture | |
| Sol_Mar | AAT GAT ACG GCG ACC ACC GAG ATC TAC ACT CTT TCC CTA CAC GAC GCT CTT CCG ATC TCG GGG ACT TAT CAG CCA ACC |
| Sol_Mar_1b | AAT GAT ACG GCG ACC ACC GAG ATC TAC ACT CTT TCC CTA CAC GAC GCT CTT CCG ATC T**T**C GGG GAC TTA TCA GCC AAC C |
| Sol_Mar_4b | AAT GAT ACG GCG ACC ACC GAG ATC TAC ACT CTT TCC CTA CAC GAC GCT CTT CCG ATC T**GA TA**C GGG GAC TTA TCA GCC AAC C |
| Sol_Mar_5b | AAT GAT ACG GCG ACC ACC GAG ATC TAC ACT CTT TCC CTA CAC GAC GCT CTT CCG ATC T**AT CTA** CGG GGA CTT ATC AGC CAA CC |
| Forward sequencing primers containing a different barcode | |
| Sol_API_tagged_1 (1) | CAA GCA GAA GAC GGC ATA CGA GAT ATC GTA CGG TGA CTG GAG TTC AGA CGT GTG CTC TTC CGA TCT GTC AAT GAT GGC CGG TGG ATT TGT G |
| Sol_API_tagged_2 (2) | CAA GCA GAA GAC GGC ATA CGA GAT ACT ATC TGG TGA CTG GAG TTC AGA CGT GTG CTC TTC CGA TCT CGT CCA TGA TGG CCG GTG GAT TTG TG |
| Sol_API_tagged_3 (3) | CAA GCA GAA GAC GGC ATA CGA GAT TAG CGA GTG TGA CTG GAG TTC AGA CGT GTG CTC TTC CGA TCT ACA GTC CCA TGA TGG CCG GTG GAT TTG TG |
| Sol_API_tagged_5 (1) | CAA GCA GAA GAC GGC ATA CGA GAT TCA TCG AGG TGA CTG GAG TTC AGA CGT GTG CTC TTC CGA TCT GTC AAT GAT GGC CGG TGG ATT TGT G |
| Sol_API_tagged_6 (2) | CAA GCA GAA GAC GGC ATA CGA GAT CGT GAG TGG TGA CTG GAG TTC AGA CGT GTG CTC TTC CGA TCT CGT CCA TGA TGG CCG GTG GAT TTG TG |
| Sol_API_tagged_8 (4) | CAA GCA GAA GAC GGC ATA CGA GAT GAC ACC GTG TGA CTG GAG TTC AGA CGT GTG CTC TTC CGA TCT TAG TGG ATG ATG GCC GGT GGA TTT GTG |
| Sol_AP1_tagged_10 (2) | CAA GCA GAA GAC GGC ATA CGA GAT CGT TAC TAG TGA CTG GAG TTC AGA CGT GTG CTC TTC CGA TCT CGT CCA TGA TGG CCG GTG GAT TTG TG |
| Sol_AP1_tagged_11 (3) | CAA GCA GAA GAC GGC ATA CGA GAT AGA GTC ACG TGA CTG GAG TTC AGA CGT GTG CTC TTC CGA TCT ACA GTC CCA TGA TGG CCG GTG GAT TTG TG |
| Sol_AP1_tagged_12 (4) | CAA GCA GAA GAC GGC ATA CGA GAT TAC GAG ACG TGA CTG GAG TTC AGA CGT GTG CTC TTC CGA TCT TAG TGG ATG ATG GCC GGT GGA TTT GTG |
| Sol-AP1-tagged -57 (1) | CAA GCA GAA GAC GGCATA CGA GAT AAG TAG AGG TGA CTG GAG TTC AGA CGT GTG CTC TTC CGA TCT GTC AAT GAT GGC CGG TGG ATT TGT G |
| Sol_API_tagged_90 (1) | CAA GCA GAA GAC GGC ATA CGA GAT TGT CAA GTG TGA CTG GAG TTC AGA CGT GTG CTC TTC CGA TCT GTC AAT GAT GGC CGG TGG ATT TGT G |
| Sol-AP1-tagged -100 (2) | CAA GCA GAA GAC GGC ATA CGA GAT ACA CGA TCG TGA CTG GAG TTC AGA CGT GTG CTC TTC CGA TCT CGT CCA TGA TGG CCG GTG GAT TTG TG |
| Sol_API_tagged_210 (2) | CAA GCA GAA GAC GGC ATA CGA GAT AGC ATA CAG TGA CTG GAG TTC AGA CGT GTG CTC TTC CGA TCT CGT CCA TGA TGG CCG GTG GAT TTG TG |
| Sol_API_tagged_240 (3) | CAA GCA GAA GAC GGC ATA CGA GAT TGC TAC GCG TGA CTG GAG TTC AGA CGT GTG CTC TTC CGA TCT ACA GTC CCA TGA TGG CCG GTG GAT TTG TG |
| Sol_API_tagged_318 (4) | CAA GCA GAA GAC GGC ATA CGA GAT AGT CTA CAG TGA CTG GAG TTC AGA CGT GTG CTC TTC CGA TCT TAG TGG ATG ATG GCC GGT GGA TTT GTG |
| Sol_API_tagged_327 (1) | CAA GCA GAA GAC GGC ATA CGA GAT CTC ATG CAG TGA CTG GAG TTC AGA CGT GTG CTC TTC CGA TCT GTC AAT GAT GGC CGG TGG ATT TGT G |
| Sol_API_tagged_360 (2) | CAA GCA GAA GAC GGC ATA CGA GAT AGT TCG GAG TGA CTG GAG TTC AGA CGT GTG CTC TTC CGA TCT CGT CCA TGA TGG CCG GTG GAT TTG TG |
| Sol-AP1-tagged -373 (4) | CAA GCA GAA GAC GGC ATA CGA GAT CAT GAT CGG TGA CTG GAG TTC AGA CGT GTG CTC TTC CGA TCT TAG TGG ATG ATG GCC GGT GGA TTT GTG |
| Sol-AP1-tagged -473 (3) | CAA GCA GAA GAC GGC ATA CGA GAT CGC GCG GTG TGA CTG GAG TTC AGA CGT GTG CTC TTC CGA TCT ACA GTC CCA TGA TGG CCG GTG GAT TTG TG |
| Sol_API_tagged_521 (4) | CAA GCA GAA GAC GGC ATA CGA GAT GAC CTG CAG TGA CTG GAG TTC AGA CGT GTG CTC TTC CGA TCT TAG TGG ATG ATG GCC GGT GGA TTT GTG |
| Sol_API_tagged_540 (1) | CAA GCA GAA GAC GGC ATA CGA GAT TGA GAC TTG TGA CTG GAG TTC AGA CGT GTG CTC TTC CGA TCT GTC AAT GAT GGC CGG TGG ATT TGT G |
| Sol-AP1-tagged -651 (2) | CAA GCA GAA GAC GGC ATA CGA GAT GCC GAT GTG TGA CTG GAG TTC AGA CGT GTG CTC TTC CGA TCT CGT CCA TGA TGG CCG GTG GAT TTG TG |
| Sol_API_tagged_666 (2) | CAA GCA GAA GAC GGC ATA CGA GAT TAC GTA CCG TGA CTG GAG TTC AGA CGT GTG CTC TTC CGA TCT CGT CCA TGA TGG CCG GTG GAT TTG TG |
| Sol_API_tagged_696 (3) | CAA GCA GAA GAC GGC ATA CGA GAT CAG TTC ATG TGA CTG GAG TTC AGA CGT GTG CTC TTC CGA TCT ACA GTC CCA TGA TGG CCG GTG GAT TTG TG |
| Sol_API_tagged_717 (4) | CAA GCA GAA GAC GGC ATA CGA GAT TCC CTA TAG TGA CTG GAG TTC AGA CGT GTG CTC TTC CGA TCT TAG TGG ATG ATG GCC GGT GGA TTT GTG |
| Sol_API_tagged_730 (1) | CAA GCA GAA GAC GGC ATA CGA GAT GTC CGA TCG TGA CTG GAG TTC AGA CGT GTG CTC TTC CGA TCT GTC AAT GAT GGC CGG TGG ATT TGT G |
| Sol_API_tagged_787 (2) | CAA GCA GAA GAC GGC ATA CGA GAT GGT TCA ACG TGA CTG GAG TTC AGA CGT GTG CTC TTC CGA TCT CGT CCA TGA TGG CCG GTG GAT TTG TG |
| Sol_API_tagged_881 (3) | CAA GCA GAA GAC GGC ATA CGA GAT CAC GTA CTG TGA CTG GAG TTC AGA CGT GTG CTC TTC CGA TCT ACA GTC CCA TGA TGG CCG GTG GAT TTG TG |
| Sol-AP1-tagged -949 (3) | CAA GCA GAA GAC GGC ATA CGA GAT TTC CGG AGG TGA CTG GAG TTC AGA CGT GTG CTC TTC CGA TCT ACA GTC CCA TGA TGG CCG GTG GAT TTG TG |
| Sol_API_tagged_976 (4) | CAA GCA GAA GAC GGC ATA CGA GAT CGA TCA AGG TGA CTG GAG TTC AGA CGT GTG CTC TTC CGA TCT TAG TGG ATG ATG GCC GGT GGA TTT GTG |
| Sol_API_tagged_1 (1) | CAA GCA GAA GAC GGC ATA CGA GAT ATC GTA CGG TGA CTG GAG TTC AGA CGT GTG CTC TTC CGA TCT GTC AAT GAT GGC CGG TGG ATT TGT G |
| Sol_API_tagged_2 (2) | CAA GCA GAA GAC GGC ATA CGA GAT ACT ATC TGG TGA CTG GAG TTC AGA CGT GTG CTC TTC CGA TCT CGT CCA TGA TGG CCG GTG GAT TTG TG |
| Sol_API_tagged_3 (3) | CAA GCA GAA GAC GGC ATA CGA GAT TAG CGA GTG TGA CTG GAG TTC AGA CGT GTG CTC TTC CGA TCT ACA GTC CCA TGA TGG CCG GTG GAT TTG TG |
| Sol_API_tagged_5 (1) | CAA GCA GAA GAC GGC ATA CGA GAT TCA TCG AGG TGA CTG GAG TTC AGA CGT GTG CTC TTC CGA TCT GTC AAT GAT GGC CGG TGG ATT TGT G |
| Sol_API_tagged_6 (2) | CAA GCA GAA GAC GGC ATA CGA GAT CGT GAG TGG TGA CTG GAG TTC AGA CGT GTG CTC TTC CGA TCT CGT CCA TGA TGG CCG GTG GAT TTG TG |
| Sol_API_tagged_8 (4) | CAA GCA GAA GAC GGC ATA CGA GAT GAC ACC GTG TGA CTG GAG TTC AGA CGT GTG CTC TTC CGA TCT TAG TGG ATG ATG GCC GGT GGA TTT GTG |
| Sol_AP1_tagged_10 (2) | CAA GCA GAA GAC GGC ATA CGA GAT CGT TAC TAG TGA CTG GAG TTC AGA CGT GTG CTC TTC CGA TCT CGT CCA TGA TGG CCG GTG GAT TTG TG |
| Sol_AP1_tagged_11 (3) | CAA GCA GAA GAC GGC ATA CGA GAT AGA GTC ACG TGA CTG GAG TTC AGA CGT GTG CTC TTC CGA TCT ACA GTC CCA TGA TGG CCG GTG GAT TTG TG |
| Sol_AP1_tagged_12 (4) | CAA GCA GAA GAC GGC ATA CGA GAT TAC GAG ACG TGA CTG GAG TTC AGA CGT GTG CTC TTC CGA TCT TAG TGG ATG ATG GCC GGT GGA TTT GTG |
