# Supplementary material for: Genome-Wide Essentiality Analysis of Mycobacterium abscessus by Saturated Transposon Mutagenesis and Deep Sequencing
Source: mBio. 2021 Jun 15;12(3):e01049-21. doi: 10.1128/mBio.01049-21 (PMC8262987; doi:10.1128/mBio.01049-21)
Supplement: TABLE S2 [file mbio.01049-21-st002.docx]

**Table S2. Statistics of TA sites with Himar1 Tn insertions in 10 independent Tn mutant pools subjected to deep sequencing**

| **Tn pool** | **Tn DNA library** | **Total read counts** | **Mapped templates** | **Mean count** | **Non-zero mean** | **TA site hits** | **Density** | **Cumulative density in triplicates** |
| --- | --- | --- | --- | --- | --- | --- | --- | --- |
| **B1** | B1-0 | 12136516 | 5831223 | 133 | 199 | 61005 | 0.67 | 0.67 |
|  | B1-1 | 12534479 | 4820788 | 137 | 202 | 62014 | 0.68 | 0.75 |
|  | B1-2 | 12285579 | 4712908 | 135 | 203 | 60429 | 0.66 | 0.78 |
| **B2** | B2-0 | 12214248 | 4200085 | 134 | 214 | 56983 | 0.63 | 0.63 |
|  | B2-1 | 12268771 | 4706062 | 135 | 208 | 59004 | 0.65 | 0.72 |
|  | B2-2 | 12217916 | 3577065 | 134 | 199 | 61455 | 0.67 | 0.76 |
| **S1** | S1-0 | 12291436 | 7260041 | 135 | 206 | 59661 | 0.65 | 0.65 |
|  | S1-1 | 12379033 | 5316047 | 136 | 199 | 62271 | 0.68 | 0.74 |
|  | S1-2 | 12368892 | 4640391 | 136 | 207 | 59659 | 0.65 | 0.75 |
| **S2** | S2-0 | 12086727 | 3973194 | 133 | 202 | 59953 | 0.66 | 0.66 |
|  | S2-1 | 12400235 | 5269149 | 136 | 204 | 60723 | 0.67 | 0.74 |
|  | S2-2 | 12324025 | 3945349 | 135 | 194 | 63462 | 0.70 | 0.78 |
| **S3** | S3-0 | 12727825 | 3169558 | 140 | 202 | 62976 | 0.69 | 0.69 |
|  | S3-1 | 12200480 | 3791762 | 134 | 213 | 57234 | 0.63 | 0.74 |
|  | S3-2 | 12056196 | 4101951 | 132 | 197 | 61145 | 0.67 | 0.78 |
| **S4** | S4-0 | 12427516 | 6797216 | 136 | 213 | 58458 | 0.64 | 0.64 |
|  | S4-1 | 12329481 | 4371966 | 135 | 203 | 60815 | 0.67 | 0.73 |
|  | S4-2 | 12399145 | 3726798 | 136 | 214 | 57827 | 0.63 | 0.74 |
| **S5** | S5-0 | 11272791 | 3259457 | 124 | 248 | 45476 | 0.50 | 0.50 |
|  | S5-1 | 12432571 | 4439348 | 136 | 194 | 64133 | 0.70 | 0.72 |
|  | S5-2 | 14304204 | 4439348 | 157 | 230 | 62153 | 0.68 | 0.77 |
| **S6** | S6-0 | 12404113 | 3559546 | 136 | 211 | 58741 | 0.64 | 0.64 |
|  | S6-1 | 14183229 | 3893121 | 155 | 231 | 61437 | 0.67 | 0.74 |
|  | S6-2 | 11728845 | 4244727 | 129 | 233 | 50457 | 0.55 | 0.76 |
| **S7** | S7-0 | 12108990 | 4319468 | 133 | 196 | 61903 | 0.68 | 0.68 |
|  | S7-1 | 12298377 | 4782748 | 135 | 201 | 61164 | 0.67 | 0.75 |
|  | S7-2 | 12524808 | 4712042 | 137 | 204 | 61358 | 0.67 | 0.78 |
| **S8** | S8a | 12207542 | 4667967 | 134 | 210 | 58186 | 0.64 | 0.64 |
|  | S8b | 12164172 | 4429516 | 133 | 213 | 57117 | 0.63 | 0.71 |
|  | S8c | 14234400 | 2785183 | 156 | 232 | 61312 | 0.67 | 0.76 |
| **Average** |  | **12450418** | **4458134** | **136** | **209** | **59617** | **0.65** | **0.77** |
