# Supplementary material for: Genome-Wide Essentiality Analysis of Mycobacterium abscessus by Saturated Transposon Mutagenesis and Deep Sequencing
Source: mBio. 2021 Jun 15;12(3):e01049-21. doi: 10.1128/mBio.01049-21 (PMC8262987; doi:10.1128/mBio.01049-21)
Supplement: TABLE S3 [file mbio.01049-21-st003.docx]

**Table S3. Distribution of TA sites with a non-permissive motif according to the number of independent Tn mutant pools in which a Tn insertion was detected at the TA site**

| **No. of pools in which a given TA site had a Tn insertion detected** | **No. of TA sites with Tn insertion detected** | **No. of those TA sites with non-permissive motif** | **% of TA sites with non-permissive motif** |
| --- | --- | --- | --- |
| 0 | 13075 | 4525 | 34.61% |
| 1 | 1551 | 405 | 26.11% |
| 2 | 1506 | 387 | 25.70% |
| 3 | 1587 | 331 | 20.86% |
| 4 | 1890 | 392 | 20.74% |
| 5 | 1723 | 294 | 17.06% |
| 6 | 1936 | 255 | 13.17% |
| 7 | 2295 | 229 | 9.98% |
| 8 | 3046 | 233 | 7.65% |
| 9 | 4450 | 177 | 3.98% |
| 10 | 58181 | 197 | 0.34% |
