# Supplementary material for: Genome-Wide Essentiality Analysis of Mycobacterium abscessus by Saturated Transposon Mutagenesis and Deep Sequencing
Source: mBio. 2021 Jun 15;12(3):e01049-21. doi: 10.1128/mBio.01049-21 (PMC8262987; doi:10.1128/mBio.01049-21)
Supplement: TABLE S4 [file mbio.01049-21-st004.docx]

**Table S4. Essential Mab genes having homology with essential genes of both Mtb H37Rv and MAH MAC109**

| **Name of gene** | **Description** |
| --- | --- |
| MAB_0001 | Chromosomal replication initiator protein DnaA |
| MAB_0006 | DNA gyrase (subunit B) GyrB (DNA topoisomerase) |
| MAB_0019 | DNA gyrase (subunit A) GyrA (DNA topoisomerase) |
| MAB_0033c | Probable serine/threonine-protein kinase PknB |
| MAB_0170 | UDP-galactopyranose mutase Glf |
| MAB_0173 | Prenyltransferase family protein UbiA |
| MAB_0178 | Hypothetical cutinase precursor |
| MAB_0179 | Probable fatty-acid-CoA ligase FadD |
| MAB_0180 | Polyketide synthase PKS13 |
| MAB_0185c | Probable arabinosyltransferase B |
| MAB_0190c | hypothetical protein |
| MAB_0305 | Putative aminotransferase |
| MAB_0323c | Putative cobyric acid synthase CobQ2 |
| MAB_0491 | Probable DNA polymerase III, delta' subunit |
| MAB_0650 | 60 kDa chaperonin 2 (Protein Cpn60 2) GroEL |
| MAB_0713 | Probable amidophosphoribosyltransferase PurF |
| MAB_1234c | Probable pantothenate kinase CoaA |
| MAB_1236 | Probable serine or glycine hydroxymethyltransferase |
| MAB_1433 | Arginyl-tRNA synthetase ArgS |
| MAB_1436 | Probable threonine synthase ThrC |
| MAB_1449 | ATP synthase B chain AtpF |
| MAB_1450 | ATP synthase delta chain AtpH |
| MAB_1451 | ATP synthase alpha chain AtpA |
| MAB_1452 | ATP synthase gamma subunit AtpG |
| MAB_1457 | Probable UDP-N-acetylglucosamine 1-carboxyvinyltransferase MurA |
| MAB_1512 | Probable fatty acid synthase Fas |
| MAB_1560 | Probable ABC transporter (macrolide-transport) ATP-binding protein |
| MAB_1583 | ATP-dependent Clp protease ATP-binding subunit ClpX |
| MAB_1603 | Valyl-tRNA synthetase |
| MAB_1604 | Probable folylpolyglutamate synthase FolC |
| MAB_1621 | Probable nicotinate-nucleotide adenylyltransferase |
| MAB_1676 | Probable undecaprenyl pyrophosphate synthetase |
| MAB_1702 | Glycyl-TRNA synthetase GlyS |
| MAB_1708 | DNA primase DnaG |
| MAB_1877c | 3-oxoacyl-[acyl-carrier-protein] synthase 1 KasA |
| MAB_1880c | hypothetical protein |
| MAB_1933c | Probable glutamine synthetase, type I GlnA1 |
| MAB_1960c | Probable asparagine synthetase AsnB |
| MAB_1984 | Probable 1-acylglycerol-3-phosphate O-acyltransferase |
| MAB_1991c | hypothetical protein |
| MAB_2001 | UDP-N-acetylmuramyl-tripeptide synthetase MurE |
| MAB_2006 | UDP-N-acetylglucosamine--N-acetylmuramyl- (pentapeptide) pyrophosphoryl-undecaprenol N-acetylglucosamine transferase MurG |
| MAB_2007 | UDP-N-acetylmuramate--L-alanine ligase MurC |
| MAB_2012 | Hypothetical protein |
| MAB_2017 | Hypothetical immunogenic protein antigen 84 |
| MAB_2104c | Probable dihydroorotate dehydrogenase |
| MAB_2296 | 30S ribosomal protein S1 |
| MAB_2335 | Phenylalanyl-tRNA synthetase beta chain PheT |
| MAB_2354 | Tyrosyl-tRNA synthetase TyrS |
| MAB_2360 | Probable inorganic polyphosphate/ATP-NAD kinase |
| MAB_2364 | CTP synthase PyrG |
| MAB_2643c | Tryptophan synthase, alpha subunit TrpA |
| MAB_2644c | Tryptophan synthase, beta subunit TrpB |
| MAB_2647c | Anthranilate synthase component I TrpE |
| MAB_2696c | DNA polymerase III alpha subunit |
| MAB_2705c | Isoleucyl-tRNA synthetase IleS |
| MAB_2721c | Ferrochelatase (Protoheme ferro-lyase) |
| MAB_2722c | Enoyl-(acyl-carrier-protein) reductase (NADH) |
| MAB_2730 | Probable aconitate hydratase Acn |
| MAB_2748c | Hypothetical protein |
| MAB_2749c | Putative FeS assembly protein SufB |
| MAB_2759 | Probable transketolase |
| MAB_2796c | Putative riboflavin biosynthesis protein RibA2 |
| MAB_2813c | Putative primosomal protein N' (ATP-dependent helicase) PriA |
| MAB_2820c | Probable S-adenosylmethionine synthetase (Methionine adenosyltransferase) |
| MAB_2827c | Carbamoyl-phosphate synthase large chain CarB |
| MAB_2828c | Carbamoyl-phosphate synthase, small chain CarA |
| MAB_2841c | 3-dehydroquinate synthase |
| MAB_2851c | Alanyl-tRNA synthetase |
| MAB_2865c | Aspartyl-tRNA synthetase AspS |
| MAB_2872c | Histidyl-tRNA synthetase |
| MAB_2898c | Threonyl-tRNA synthetase |
| MAB_2978 | Hypothetical protein |
| MAB_2984c | Putative chlorite dismutase |
| MAB_2985c | Protoporphyrinogen oxidase HemY' |
| MAB_2986c | Probable uroporphyrinogen decarboxylase HemE |
| MAB_2990c | Probable 1-deoxy-d-xylulose-5-phosphate synthase DXS |
| MAB_3009 | Probable RNA polymerase sigma factor RpoD |
| MAB_3074c | Probable cell division protein FtsK |
| MAB_3106c | Bifunctional protein polyribonucleotide nucleotidyltransferase GpsI |
| MAB_3109c | Probable bifunctional FAD synthetase/riboflavin biosynthesis protein RibF |
| MAB_3131c | Translation initiation factor IF-2 InfB |
| MAB_3140c | Prolyl-tRNA synthetase ProS |
| MAB_3171c | 1-deoxy-D-xylulose-5-phosphate reductoisomerase |
| MAB_3188c | Uridylate kinase PyrH |
| MAB_3269c | Hypothetical protein |
| MAB_3286c | Probable D-alanine-D-alanine ligase DdlA |
| MAB_3298c | Glutamyl-tRNA synthetase GltX |
| MAB_3334c | Aspartyl/glutamyl-tRNA amidotransferase, B subunit GatB |
| MAB_3341c | Glutamyl-tRNA(Gln) amidotransferase subunit A GatA |
| MAB_3345c | DNA ligase (polydeoxyribonucleotide synthase NAD+) |
| MAB_3404c | Probable ribonucleoside-diphosphate reductase beta subunit |
| MAB_3413c | Ribonucleoside-diphosphate reductase alpha subunit |
| MAB_3414c | Protein NrdI |
| MAB_3415c | Glutaredoxin-like protein NrdH |
| MAB_3473c | SsrA-binding protein |
| MAB_3478c | Peptide chain release factor 2 PrfB |
| MAB_3594c | Probable thymidylate kinase Tmk |
| MAB_3601c | Probable mannose-6-phosphate isomerase ManA |
| MAB_3603c | Probable phosphomannomutase |
| MAB_3611c | Putative sugar-phosphate nucleotidyl transferase |
| MAB_3612c | Putative dTDP-rhamnosyltransferase |
| MAB_3613c | Possible DTDP-rhamnose modification protein RmlD |
| MAB_3631 | Probable propionyl-CoA carboxylase beta chain 5 AccD5 |
| MAB_3643 | Probable bifunctional protein acetyl-/propionyl-CoA carboxylase (alpha chain) AccA3 |
| MAB_3683c | Tryptophanyl-tRNA synthetase TrpS |
| MAB_3718c | GMP synthase [glutamine-hydrolyzing] |
| MAB_3721c | Probable inosine-5'-monophosphate dehydrogenase GuaB2 |
| MAB_3743c | Glucosamine--fructose-6-phosphate aminotransferase |
| MAB_3750c | Phosphoglucosamine mutase MrsA |
| MAB_3771c | 30S ribosomal protein S4 |
| MAB_3773c | 30S ribosomal protein S13 |
| MAB_3779 | DTDP-glucose 4,6-dehydratase RmlB |
| MAB_3784c | Preprotein translocase secY subunit |
| MAB_3797c | 50S ribosomal protein L6 |
| MAB_3798c | 30S ribosomal protein S8 |
| MAB_3813c | 50S ribosomal protein L16 |
| MAB_3848c | Elongation factor Tu (EF-Tu) |
| MAB_3849c | Elongation factor G (EF-G) |
| MAB_3868c | DNA-directed RNA polymerase beta' chain |
| MAB_3869c | DNA-directed RNA polymerase beta chain |
| MAB_3978c | Probable glutamate-1-semialdehyde aminotransferase |
| MAB_3990c | Probable delta-aminolevulinic acid dehydratase HemB (porphobilinogen synthase) |
| MAB_3993c | Glutamyl-tRNA reductase HemA |
| MAB_3996 | Hypothetical protein |
| MAB_4113 | Glucose-1-phosphate thymidylyltransferase |
| MAB_4249c | Adenylosuccinate synthetase PurA |
| MAB_4273c | Chaperone protein DnaK (Hsp 70) |
| MAB_4472 | Hypothetical protein |
| MAB_4474 | Hypothetical protein |
| MAB_4480c | Putative glycosyl transferase |
| MAB_4895c | Replicative DNA helicase [Contains: EndonucleasePI-MtuHIP (Mtu DnaB intein)] |
| MAB_4907 | Myo-inositol-1-phosphate synthase |
| MAB_4923c | Leucyl-tRNA synthetase |
| MAB_4934c | Poly(A) polymerase PcnA |
