# Supplementary material for: Genome-Wide Essentiality Analysis of Mycobacterium abscessus by Saturated Transposon Mutagenesis and Deep Sequencing
Source: mBio. 2021 Jun 15;12(3):e01049-21. doi: 10.1128/mBio.01049-21 (PMC8262987; doi:10.1128/mBio.01049-21)
Supplement: TABLE S5 [file mbio.01049-21-st005.docx]

**Table S5. Essential Mab genes having homology with essential genes in either Mtb H37Rv or MAH MAC109, but not both**

| **Name of gene** | **Description** |
| --- | --- |
|  | **Mab genes having orthologs in Mtb H37Rv, but not MAH MAC109** |
| MAB_0002 | DNA polymerase III, beta subunit DnaN |
| MAB_0032 | Glutamine amidotransferase of anthranilate synthase component II PabA |
| MAB_0118c | Probable superoxide dismutase (Mn) |
| MAB_0153 | Seryl-tRNA synthetase SerS |
| MAB_0171 | Galactofuranosyl transferase |
| MAB_0181 | Propionyl-CoA carboxylase, beta subunit PccB |
| MAB_0186c | Probable arabinosyltransferase A |
| MAB_0192c | Probable oxidoreductase |
| MAB_0203c | Probable o-antigen/lipopolysaccharide transport ATP-binding protein ABC transporter RfbE |
| MAB_0204c | Hypothetical protein |
| MAB_0266c | Probable prephenate dehydrogenase TyrA |
| MAB_0309 | DNA polymerase III, subunit gamma/tau |
| MAB_0324c | Putative Mur ligase family protein |
| MAB_0441 | Hypothetical protein |
| MAB_0489 | DNA topoisomerase I (Omega-protein) |
| MAB_0503 | Probable UDP-glucose 4-epimerase GalE1 |
| MAB_0518c | Inorganic pyrophosphatase |
| MAB_0521 | Putative tRNA(Ile)-lysidine synthase |
| MAB_0541 | Probable pantoate--beta-alanine ligase PanC |
| MAB_0544 | Lysyl-trna synthetase LysS |
| MAB_0546 | Probable ATP-dependent Clp protease ATP-binding subunit |
| MAB_0571 | Cysteinyl-tRNA synthetase |
| MAB_0682 | Probable phosphoribosylamine--glycine ligase GarS |
| MAB_0687 | Adenylosuccinate lyase |
| MAB_0689 | Probable phosphoribosylaminoimidazole-succinocarboxamide synthase PurC |
| MAB_0697 | Hypothetical protein |
| MAB_0698 | Probable phosphoribosylformylglycinamidine synthase I |
| MAB_0707 | Probable phosphoribosylformylglycinamidine synthase II PurL |
| MAB_0728 | Probable phosphoribosylformylglycinamidine cyclo-ligase PurM |
| MAB_0730c | Conserved hypothetical protein (glycine cleavageT-protein aminomethyl transferase?) |
| MAB_0928c | Putative phosphoserine aminotransferase |
| MAB_1064 | Probable bifunctional purine biosynthesis protein PurH |
| MAB_1077 | Probable two component sensor kinase MprB |
| MAB_1086 | Putative UTP-glucose-1-phosphate uridylyltransferase GalU |
| MAB_1128c | Methionyl-tRNA synthetase MetG |
| MAB_1139 | 4-(cytidine 5'-diphospho)-2-C-methyl-D-erythritol kinase |
| MAB_1142c | Probable peptidyl-tRNA hydrolase PTH |
| MAB_1147c | Probable ribose-phosphate pyrophosphokinase RppK (PRPP synthetase) |
| MAB_1148c | Probable UDP-N-acetylglucosamine pyrophosphorylase GlmU |
| MAB_1237 | Possible acyl-[acyl-carrier protein] desaturase DesA2 |
| MAB_1257 | 4-hydroxy-3-methylbut-2-enyl diphosphate reductase |
| MAB_1315 | Putative lipoprotein LpqW |
| MAB_1327 | Probable ferredoxin FdxC |
| MAB_1335c | 2,3,4,5-tetrahydropyridine-2,6-dicarboxylate N-succinyltransferase |
| MAB_1336 | Probable succinyl-diaminopimelate desuccinylase |
| MAB_1366c | Putative Mrp homolog protein |
| MAB_1435 | Probable homoserine dehydrogenase |
| MAB_1437 | Probable homoserine kinase ThrB |
| MAB_1438 | Probable transcription termination factor Rho |
| MAB_1442 | Probable peptide chain release factor 1 (RF-1) |
| MAB_1445 | Putative undecaprenyl-phosphate alpha-N-acetylglucosaminyltransferase |
| MAB_1447 | ATP synthase A chain AtpB |
| MAB_1448 | ATP synthase C chain AtpE |
| MAB_1453 | ATP synthase beta subunit AtpD |
| MAB_1533c | Probable oligoribonuclease |
| MAB_1581 | ATP-dependent Clp protease, proteolytic subunit 2 |
| MAB_1582 | ATP-dependent Clp protease, proteolytic subunit 1 |
| MAB_1612 | Probable GTP1/Obg-family GTP-binding protein |
| MAB_1633 | Putative DNA polymerase III delta subunit |
| MAB_1878c | Acyl carrier protein |
| MAB_1879c | Putative malonyl CoA-ACP transacylase FabD |
| MAB_1916c | 3-methyl-2-oxobutanoate hydroxymethyltransferase |
| MAB_1970 | Anthranilate phosphoribosyltransferase TrpD |
| MAB_1976 | Conserved hypothetical protein (glycosyl transferase?) |
| MAB_1987 | 3-deoxy-7-phosphoheptulonate synthase |
| MAB_2002 | UDP-N-acetylmuramoyl-tripeptide--D-alanyl-D- alanine ligase MurF |
| MAB_2003 | Phospho-N-acetylmuramoyl-pentapeptide- transferaseMurX |
| MAB_2004 | UDP-N-acetylmuramoylalanine--D-glutamate ligase MurD |
| MAB_2008 | Putative cell division protein FtsQ |
| MAB_2321 | Translation initiation factor IF-3 InfC |
| MAB_2323 | 50S ribosomal protein L20 |
| MAB_2334 | Phenylalanyl-tRNA synthetase alpha chain PheS |
| MAB_2398 | Putative phosphatidylglycerophosphate synthase PgsA2 |
| MAB_2645c | Indole-3-glycerol phosphate synthase TrpC |
| MAB_2723c | 3-oxoacyl-[ACP] reductase MabA |
| MAB_2777c | Probable triosephosphate isomerase TpiA |
| MAB_2781c | Hypothetical protein |
| MAB_2795c | Probable 6,7-dimethyl-8-ribityllumazine synthase(riboflavin synthase beta chain) |
| MAB_2808c | Probable bifunctional riboflavin biosynthesis protein RibG |
| MAB_2821c | Putative pantothenate metabolism flavoprotein |
| MAB_2826c | Orotidine 5'-phosphate decarboxylase PyrF |
| MAB_2830c | Dihydroorotase PyrC |
| MAB_2831c | Aspartate carbamoyltransferase |
| MAB_2840c | 3-dehydroquinate dehydratase AroQ |
| MAB_2894c | Probable phosphatidylinositol alpha-mannosyltransferase |
| MAB_3043c | Probable diaminopimelate epimerase DapF |
| MAB_3071c | Probable PGP synthase PgsA3 (phosphatidylglycerophosphate synthase) |
| MAB_3084c | 4-hydroxy-tetrahydrodipicolinate synthase |
| MAB_3135c | Transcription elongation protein NusA |
| MAB_3169c | 4-hydroxy-3-methylbut-2-en-1-yl diphosphate synthase |
| MAB_3186c | Phosphatidate cytidylyltransferase CdsA |
| MAB_3187c | Ribosome recycling factor (Ribosome-releasing factor |
| MAB_3195c | Elongation factor Ts (EF-Ts) |
| MAB_3196c | 30s ribosomal protein S2 |
| MAB_3223c | Probable signal peptidase I LepB |
| MAB_3237c | Signal recognition particle protein Ffh |
| MAB_3241c | Cell division protein FtsY homolog |
| MAB_3258c | Hypothetical protein |
| MAB_3268c | Hypothetical protein |
| MAB_3284c | Probable thiamine-monophosphate kinasE ThiL |
| MAB_3304c | D-3-phosphoglycerate dehydrogenase SerA |
| MAB_3362c | Electron transfer flavoprotein alpha-subunit FixB |
| MAB_3365 | Hypothetical protein |
| MAB_3378c | Conserved hypothetical protein (transferase?) |
| MAB_3388c | Probable phosphoserine phosphatase SerB2 |
| MAB_3546 | 3-phosphoshikimate 1-carboxyvinyltransferase |
| MAB_3655c | Probable glycerol-3-phosphate dehydrogenase |
| MAB_3692c | Probable bifunctional protein FolD |
| MAB_3732c | 10 kDa chaperonin GroES |
| MAB_3780 | DTDP-4-dehydrorhamnose 3,5-epimerase RmlC |
| MAB_3795c | 30S ribosomal protein S5 |
| MAB_3796c | 50S ribosomal protein L18 |
| MAB_3805c | 50S ribosomal protein L5 |
| MAB_3806c | 50S ribosomal protein L24 |
| MAB_3807c | 50S ribosomal protein L14 |
| MAB_3811c | 30S ribosomal protein S17 |
| MAB_3814c | 30S ribosomal protein S3 |
| MAB_3815c | 50S ribosomal protein L22 |
| MAB_3816c | 30S ribosomal protein S19 |
| MAB_3850c | 30S ribosomal protein S7 |
| MAB_3851c | 30S ribosomal protein S12 |
| MAB_3892c | 50S ribosomal protein L1 |
| MAB_3958 | 1,4-dihydroxy-2-naphthoate polyprenyltransferase |
| MAB_3992c | Porphobilinogen deaminase HemC |
| MAB_4066c | UDP-N-acetylenolpyruvoylglucosamine reductase MurB |
| MAB_4260c | Orotate phosphoribosyltransferase |
| MAB_4473c | Acyltransferase |
| MAB_4475 | Hypothetical protein |
| MAB_4876c | Hypothetical protein |
| MAB_4936 | Hypothetical protein |
| MAB_4940 | Thioredoxin reductase TrxB |
| MAB_4942 | N-acetylmuramoyl-L-alanine amidase CwlM |
| MAB_4949c | Probable chromosome partitioning protein ParB |
| MAB_4950c | Putative chromosome partitioning protein/ cobyrinic acid a,c-diamide synthase |
|  | **Mab genes having orthologs in MAH MAC109 but not Mtb H37Rv** |
| MAB_0502c | Hypothetical protein |
| MAB_4937 | Hypothetical protein |
| MAB_2207 | Possible polyprenol phosphate mannosyl transferase 1 Ppm1 |
| MAB_3580c | Preprotein translocase secA 1 subunit |
| MAB_3511c | Probable DNA helicase II homolog UvrD2 |
| MAB_1920 | Probable glutamine synthetase |
| MAB_2429c | Probable NADH dehydrogenase NDH |
| MAB_2000 | Probable penicillin-binding membrane protein PbpB |
| MAB_2874 | Probable peptidyl-prolyl cis-trans isomerase |
| MAB_4508 | Putative membrane protein MmpL |
| MAB_3091c | Thymidylate synthase ThyA |
