# Supplementary material for: Genome-Wide Essentiality Analysis of Mycobacterium abscessus by Saturated Transposon Mutagenesis and Deep Sequencing
Source: mBio. 2021 Jun 15;12(3):e01049-21. doi: 10.1128/mBio.01049-21 (PMC8262987; doi:10.1128/mBio.01049-21)
Supplement: TABLE S6 [file mbio.01049-21-st006.docx]

**Table S6. Essentiality analysis of Mab genes having homology with Mtb genes involved in type VII secretion (T7S) systems**

| **T7S system** | **Gene name** | **Gene product** | **Mtb gene** | **Call** | **Mab gene** | **Description of Mab gene** | **Call** |
| --- | --- | --- | --- | --- | --- | --- | --- |
| ESX-3 | eccA3 | AAA+ ATPase | Rv0282 | ES | MAB_2234c | Conserved hypothetical protein (AAA ATPase?) | NE |
|  | eccB3 | Transmembrane protein | Rv0283 | ES | MAB_2233c | hypothetical protein | NE |
|  | eccC3 | FtsK/SpoIIIE-like transmembrane protein | Rv0284 | ES | MAB_2232c | Putative FtsK/SpoIIIE family protein | NE |
|  | eccD3 | Transmembrane protein | Rv0290 | ES | MAB_2226c | hypothetical protein | NE |
|  | eccE3 | Transmembrane protein | Rv0292 | ES | MAB_2224c | hypothetical protein | NE |
|  | eccF3 | Subtilisin-like serine protease (Mycosin) | Rv0291 | ES | MAB_2225c | Probable peptidase | NE |
|  | espG3 | Soluble protein | Rv0289 | ES | MAB_2227c | hypothetical protein | NE |
| ESX-4 | eccA | AAA+ ATPase |  |  |  |  | NE |
|  | eccB4 | Transmembrane protein | Rv3450c | NE | MAB_3759c | hypothetical protein | NE |
|  | eccC4 | FtsK/SpoIIIE-like transmembrane protein | Rv3447c | NE | MAB_3756c | Putative FtsK/SpoIIIE family protein | NE |
|  | eccD4 | Transmembrane protein | Rv3448 | NE | MAB_3757^*^ | Conserved hypothetical protein | NE |
|  | eccE4 | Transmembrane protein |  |  | MAB_3760^*^ | hypothetical protein | NE |
|  | eccF4 | Subtilisin-like serine protease (Mycosin) | Rv3449 | NE | MAB_3758 | Putative protease | NE |
|  | espG | Soluble protein |  |  |  |  |  |

Note: The table was referenced from Bitter W, Houben EN, Bottai D, Brodin P, Brown EJ, Cox JS, et al. Systematic genetic nomenclature for type VII secretion systems. PLoS pathogens. 2009;5(10):e1000507. *: The information was obtained from Laencina L, Dubois V, Le Moigne V, Viljoen A, Majlessi L, Pritchard J, et al. Identification of genes required for Mycobacterium abscessus growth in vivo with a prominent role of the ESX-4 locus. Proceedings of the National Academy of Sciences of the United States of America. 2018;115(5):E1002. ES: essential; GD: growth defect when mutated; GA: growth advantage when mutated; NE: non-essential
