# Supplementary material for: Genome-Wide Essentiality Analysis of Mycobacterium abscessus by Saturated Transposon Mutagenesis and Deep Sequencing
Source: mBio. 2021 Jun 15;12(3):e01049-21. doi: 10.1128/mBio.01049-21 (PMC8262987; doi:10.1128/mBio.01049-21)
Supplement: TABLE S7 [file mbio.01049-21-st007.docx]

**Table S7. Essentiality analysis and homology comparison of Mab genes with homology to β-lactamases**

| **Name of gene** | **Mab genes having orthologs in Mtb H37Rv** | **Call** |
| --- | --- | --- |
| MAB_0330 | Beta-lactamase | NE |
| MAB_2875 | Beta-lactamase, Bla_Mab_ | NE |
|  | **Mab genes having orthologs in Mtb H37Rv and MAH MAC109** |  |
| MAB_2314c | Beta-lactamase-like | GA |
| MAB_1312 | Beta-lactamase-like | NE |
| MAB_4231 | Beta-lactamase-like protein | NE |
| MAB_4755c | Conserved hypothetical protein (beta-lactamase?) | NE |
| MAB_0696c | Conserved hypothetical protein (beta-lactamase?) | NE |
| MAB_0414 | Possible hydrolase (beta-lactamase-like) | NE |
| MAB_2833 | Probable beta-lactamase | GA |
| MAB_1870 | Putative beta-lactamase | NE |
| MAB_1387 | Putative esterase/lipase/beta-lactamase | NE |
| MAB_1386 | Putative esterase/lipase/beta-lactamase | NE |
| MAB_4006 | Putative lipase/esterase/beta-lactamase | NE |
|  | **Mab genes having orthologs in MAH MAC109** |  |
| MAB_4800 | Putative beta-lactamase | NE |
|  | **Mab genes having no orthologs in Mtb H37Rv or MAH MAC109** |  |
| MAB_2179 | Beta-lactamase/esterase | NE |
| MAB_4805 | Beta-lactamase-like hypothetical protein | NE |
| MAB_4947 | Beta-lactamase-like protein | NE |
| MAB_1114 | Putative metallo-beta-lactamase superfamily | NE |

Note: ES: essential; GD: growth defect when mutated; GA: growth advantage when mutated; NE: non-essential
