# Supplementary material for: Genome-Wide Essentiality Analysis of Mycobacterium abscessus by Saturated Transposon Mutagenesis and Deep Sequencing
Source: mBio. 2021 Jun 15;12(3):e01049-21. doi: 10.1128/mBio.01049-21 (PMC8262987; doi:10.1128/mBio.01049-21)
Supplement: TABLE S8 [file mbio.01049-21-st008.docx]

**Table S8. Essential Mab genes that lack homologs in *M. chelonae* type strain CCUG 47445**

| **Name of gene** | **Description** |
| --- | --- |
| MAB_4955c | 50S ribosomal protein L34 |
| MAB_1878c | acyl carrier protein |
| MAB_4473c | acyltransferase |
| MAB_3848c | Elongation factor Tu (EF-Tu) |
| MAB_0210 | hypothetical protein |
| MAB_0441 | hypothetical protein |
| MAB_1556 | hypothetical protein |
| MAB_2350c | Hypothetical protein |
| MAB_3624c | Hypothetical protein |
| MAB_4471 | Hypothetical protein |
| MAB_4828c | Hypothetical protein |
| MAB_4876c | hypothetical protein |
| MAB_0222c | Putative DNA-binding protein |
