# Supplementary material for: Genome-Wide Essentiality Analysis of Mycobacterium abscessus by Saturated Transposon Mutagenesis and Deep Sequencing
Source: mBio. 2021 Jun 15;12(3):e01049-21. doi: 10.1128/mBio.01049-21 (PMC8262987; doi:10.1128/mBio.01049-21)
Supplement: TEXT S1 [file mbio.01049-21-s0001.docx]

# **Genome-wide essentiality analysis of *Mycobacterium abscessus* by saturated transposon mutagenesis and deep sequencing**

Dalin Rifat, Liang Chen, Barry N. Kreiswirth, Eric L. Nuermberger

**Construction of Himar1 transposon mutant pools**

To generate a fully saturated transposon (Tn) mutant pool using the Mab ATCC 19977^T^ strain, a protocol from the published literature for constructing a Himar1 Tn mutant pool was systematically optimized with modifications [[1](#_ENREF_1), [3](#_ENREF_3)]. A single phage plaque was isolated from top agar plates by infecting *M. smegmatis* mc^2^155 with ΦmycomarT7 phage. The temperature sensitivity of the phage was checked at 30°C and 37°C before phage propagation to confirm that the phages were lytic at 30°C and lysogenic at 37°C. Mab ATCC 19977^T^ was subcultured in 7H9 broth. After the O.D._600nm_ reached 1.0 - 2.0, the culture was washed twice with 7H9 broth without Tween 80 and the bacterial pellet was suspended in MP buffer (50 mM Tris–HCl (pH 7.5), 150 mM NaCl, 10 mM MgSO_4_, 2 mM CaCl_2_, filter-sterilized using a 0.22 μm filter). The optimization included testing the impact of the following parameters: 1. MOI (ratio of the number of ΦmycomarT7 plaque-forming units (PFU) to the number of Mab colony-forming units (CFU)): 1.5X10^9^ CFU/ml of Mab ATCC 19977^T^ was infected at MOI of 10:1, 20:1, 50:1 and 130:1, respectively, in a 37°C shaker at 90 rpm for 3 hours. 2. Incubation time after transfection: 1.5X10^9^ CFU/ml of the strain was infected at MOI of 10:1 in a 37°C shaker at 90 rpm for 1, 2, 3, 4 and 5 hours, respectively. 3. Incubation status (without shaking or with shaking at different speeds): 1.5X10^9^ CFU/ml of the strain was infected with phage at MOI of 20:1 for 4 hours in a 37°C shaker standing still and shaking at 45, 90 and 180 rpm, respectively. 4. Density of Mab used in the transfection: 8.2 X 10^10^ CFU/ml of the strain was infected at MOI of 20:1 in a 37°C shaker at 180 rpm for 4 hours to compare with 1.5 X 10^9^ CFU/ml of the strain under the same conditions. The transfection was stopped by washing once with MP buffer containing 20 mM sodium citrate and 0.2% Tween-80. After centrifugation the bacterial pellet was re-suspended in the same stop solution and serial dilutions were plated on 7H11 agar plates with 50 µg/ml of kanamycin. Wild-type Mab was also plated prior to the infection. The frequencies of transposon insertion and spontaneous kanamycin resistance mutations were determined based on the number of kanamycin-resistant colonies per total number of input bacteria after 7 days of incubation at 37°C. Ten Mab Tn pools were generated using our optimized protocol. For isolation of a Tn mutant pool, the bacterial suspension after phage infection was plated without dilution on 7H11 agar plates containing 0.1% Tween 80 and 50 µg/ml of kanamycin prepared using square bioassay dishes (Corning, 245mm X 245mm). CFUs of transposon mutants and spontaneous mutants were determined for each Tn pool as described above. After scraping from the plates and suspending in 7H9 broth, the Himar1 Tn mutant pools were frozen in aliquots at -80°C for further use.

To confirm the quality of the collected mutant pools, single colonies of the individual Tn mutants were randomly collected from one of the pools and subjected to colony-PCR of the kanamycin resistance gene using primers Kan-F and Kan-R (Table S2) after boiling the colony in distilled water containing 0.05% SDS for 5 min. Genomic DNA purified from colonies was used to determine the transposon junctions, as described by Lamichhane, *et al* with modification [[2](#_ENREF_2)]. *Alu*I-digested DNA was ligated with annealed adapters of AdBTM and AdTOP (Table S2) followed by PCR amplification with primers T7-A and AP1 (Table S2) using Q5 high-fidelity 2X master mix (New England Biolabs) and the following cycles (98°C 3 min; 30 cycles of 98°C for 30 s, 60 °C for 30 s, 72°C for 1 min; 72°C for 5 min). The PCR product from each colony was examined on agarose gel followed by DNA sequencing to determine the sequence of the transposon-genome junction.

**Preparation of DNA libraries of Tn mutant pools**

DNA libraries of Tn mutant pools were prepared as previously described, with modifications [[1](#_ENREF_1), [3](#_ENREF_3)]. Prior to DNA library preparation, genomic DNA was purified from each Tn mutant pool using an established CTAB method, with modifications. First, 300 µl of frozen aliquot (about 10^10^ CFU) of a Tn pool was heat-killed on a heat block at 90°C for 20 min in 300 µl of TE buffer (10 mM Tris-HCl, 1 mM disodium EDTA, pH 8.0) after replacement of 7H9 broth by centrifugation. Then the heat-killed cell suspension was transferred into a 2 ml O-ring tube previously filled with about 20 autoclaved 2mm glass beads prior to addition of an equal amount of 10 mg/ml of lysozyme solution. The tube was vortexed 3 times for 30 s over a 1-hour interval while incubating at 37°C and then continued incubating overnight. After adding 120 µl of 5% SDS and 120 µl of 1mg/ml proteinase K solution, it was incubated at 50°C and vortexed for 30 s twice over 10 min of incubation. Following that, 120 µl of 3M NaCl and 60 µl of CTAB/NaCl solution (containing 4.1 % of NaCl, 10 % of N-cetyl-N, N, N, -trimethyl ammonium bromide (CTAB)) was added. After centrifugation 900 µl of the supernatant was transferred into a new O-ring tube. An equal amount of chloroform:isoamylalcohol (24:1) was added and mixed well by inverting the tube. After centrifugation at 13,000 rpm for 8 min the top aqueous layer (about 750 µl) was transferred into another new tube followed by addition of an equal amount of ice-cold isopropanol to precipitate the genomic DNA at -20°C for 30 min. After centrifugation at 13,000 rpm for 15 min, the DNA precipitate was subjected to washing once with 1 ml of ice-cold 70% ethanol. After the removal of the ethanol by centrifugation at 13,000 rpm for 5 min the DNA precipitate was dried at room temperature and then dissolved in 200 µl of TE buffer.

Purified genomic DNA from each Tn mutant pool was used to prepare a DNA library for deep sequencing, as described, with modifications [[1](#_ENREF_1), [3](#_ENREF_3)]. DNA shearing, end-repair and A-tailing were performed as previously described [[1](#_ENREF_1)], but the resultant DNA fragments were purified with Monarch PCR and DNA cleanup kit (New England Biolabs). About 600 ng of A-tailed DNA fragments in 30 µl of TE buffer were ligated with 2 µl of 25 µM annealed adapters (adapter 1.2 and adapter 2.2 Bar B, Table S2) using an equal amount of Blunt/TA ligase master mix (New England Biolabs) and purified as described [[1](#_ENREF_1)]. Adapter-ligated DNA fragments were used as templates to perform first PCR to enrich the transposon junctions using primers JEL_API and T7-A [[3](#_ENREF_3)] (Table S2) that replaced primer T7 used in previous protocol with better specificity. The following program was used to amplify 250 µl of PCR reaction of Q5 hot start high fidelity 2X master mix (New England Biolabs) containing 600 ng of adapter-ligated DNA fragments: 98°C 30 s; 20 cycles of 98°C 10 s, 63°C 20 s, 72°C 30 s; 72°C 2 min. The PCR products were subjected to two rounds of size-selection by SPRIselect beads (Beckman) with ratio of 0.55X - 0.75X to purify 400-600-bp of PCR products according to manufacturer’s protocol. Then secondary PCR was performed using purified PCR products and a pair of sequencing primers, in which the reverse sequencing primer Sol-mar mixture consisted of four primers with four types of staggered sequences to avoid generating a low-diversity sequencing sample (Table S2). For each library, we used a forward sequencing primer containing a different barcode and one of four types of staggered sequence before the adapter sequence to avoid low-diversity samples when pooling the libraries together for deep sequencing. A 250 µl PCR reaction in Q5 hot start high fidelity 2X master mix (New England Biolabs) containing 500 ng of purified PCR products of transposon junctions was performed using the following program: 98°C for 30 s; 10 cycles of 98°C for 10 s, 69°C for 20 s, 72°C for 30 s; 72°C for 2 min; and then the PCR products were purified with the same beads using two rounds of size selection with ratio of 0.50X - 0.70X. All sequencing primers were synthesized with PAGE gel purification and checked on a bioanalyzer. Only those with impurity of <8% were chosen in our study. For each of the Tn pools, we prepared triplicate DNA libraries of ≥25 ng/µl in 20 µl of TE buffer (measured by Nanodrop) for deep sequencing.

**References**

1. Matern WM, Jenquin RL, Bader JS, Karakousis PC. Identifying the essential genes of Mycobacterium avium subsp. hominissuis with Tn-Seq using a rank-based filter procedure. Scientific reports. 2020;10(1):1095. Epub 2020/01/25. doi: 10.1038/s41598-020-57845-7. PubMed PMID: 31974396; PubMed Central PMCID: PMC6978383.

2. Lamichhane G, Zignol M, Blades NJ, Geiman DE, Dougherty A, Grosset J, et al. A postgenomic method for predicting essential genes at subsaturation levels of mutagenesis: application to Mycobacterium tuberculosis. Proceedings of the National Academy of Sciences of the United States of America. 2003;100(12):7213-8. Epub 2003/05/31. doi: 10.1073/pnas.1231432100. PubMed PMID: 12775759; PubMed Central PMCID: PMC165855.

3. Long JE, DeJesus M, Ward D, Baker RE, Ioerger T, Sassetti CM. Identifying essential genes in Mycobacterium tuberculosis by global phenotypic profiling. Methods Mol Biol. 2015;1279:79-95. Epub 2015/02/01. doi: 10.1007/978-1-4939-2398-4_6. PubMed PMID: 25636614.
